# Epigenome-wide analysis reveals functional modulators of drug sensitivity and post-treatment survival in chronic lymphocytic leukaemia

**DOI:** 10.1038/s41416-020-01117-8

**Published:** 2020-10-21

**Authors:** Timothy M. Barrow, Sirintra Nakjang, Fadhel Lafta, Kateryna Bilotkach, Laura Woodhouse, Gesa Junge, Susan J. Tudhope, Jonathan P. Wallis, Helen Marr, Scott Marshall, Nick Bown, Elaine Willmore, Gordon Strathdee

**Affiliations:** 1 Newcastle University Centre for Cancer, Biosciences Institute, Newcastle University, Newcastle upon Tyne, UK; 2 Faculty of Health Sciences & Wellbeing, University of Sunderland, Sunderland, UK; 3 Cancer Research UK Drug Discovery Unit, Translational & Clinical Research Institute, Newcastle University, Newcastle upon Tyne, UK; 4 Department of Biology, College of Science, University of Baghdad, Baghdad, Iraq; 5 School of Medical Education, Faculty of Medical Sciences, Newcastle University, Newcastle upon Tyne, UK; 6 Department of Haematology, Freeman Hospital, Newcastle upon Tyne, UK; 7 Department of Haematology City Hospitals Sunderland NHS Trust, Sunderland Royal Hospital, Sunderland, UK; 8 Northern Genetics Service, Institute of Genetic Medicine, Central Parkway, Newcastle upon Tyne, UK

**Keywords:** Genetics research, Cancer genomics, Chronic lymphocytic leukaemia, DNA methylation, Epigenomics

## Abstract

**Background:**

Chronic lymphocytic leukaemia (CLL) patients display a highly variable clinical course, with progressive acquisition of drug resistance. We sought to identify aberrant epigenetic traits that are enriched following exposure to treatment that could impact patient response to therapy.

**Methods:**

Epigenome-wide analysis of DNA methylation was performed for 20 patients at two timepoints during treatment. The prognostic significance of differentially methylated regions (DMRs) was assessed in independent cohorts of 139 and 163 patients. Their functional role in drug sensitivity was assessed in vitro.

**Results:**

We identified 490 DMRs following exposure to therapy, of which 31 were CLL-specific and independent of changes occurring in normal B-cell development. Seventeen DMR-associated genes were identified as differentially expressed following treatment in an independent cohort. Methylation of the *HOXA4*, *MAFB* and *SLCO3A1* DMRs was associated with post-treatment patient survival, with *HOXA4* displaying the strongest association. Re-expression of HOXA4 in cell lines and primary CLL cells significantly increased apoptosis in response to treatment with fludarabine, ibrutinib and idelalisib.

**Conclusion:**

Our study demonstrates enrichment for multiple CLL-specific epigenetic traits in response to chemotherapy that predict patient outcomes, and particularly implicate epigenetic silencing of *HOXA4* in reducing the sensitivity of CLL cells to therapy.

## Background

Chronic lymphocytic leukaemia (CLL) is marked by a highly variable clinical course, with some patients displaying indolent disease for many years, while others require immediate therapeutic intervention and have significantly inferior outcomes. One of the most informative prognostic biomarkers is somatic hypermutation levels of the immunoglobulin heavy-chain variable region (*IGHV*), informing upon the cell of origin of the disease, with unmutated *IGHV* strongly associated with shorter time to first treatment and reduced survival.^1^ Other prognostic biomarkers, including the expression of *CD38* and *ZAP70*, have also been shown to predict patient outcome.^2,3^ Chromosomal abnormalities such as del(11q) and del(17p) are associated with aggressive disease and reduced survival, but are acquired during disease progression and rarely present at diagnosis.^4^ Currently, *IGHV* mutation and del(17p) or *TP53* mutation are the only prognostic markers that inform upon the direction of treatment, and the biological mechanisms underlying treatment failure have not been elucidated.

Recent advances in the field have demonstrated the expansion of genetic subclones during the progression of CLL,^5^ including selection for resistant subclones following therapeutic intervention.^6^ However, substantially less is understood about changes in the CLL epigenome associated with disease progression and response to treatment, and how this may influence patient outcomes. Similar to the acquisition of somatic mutations, epigenetic changes in CLL occur more frequently in late-replication domains of the genome, and their acquisition appears to be stochastic.^7^ The changes observed in CLL strongly mirror those that occur during B-cell differentiation,^8^ with *IGHV*-unmutated and mutated CLL cases displaying distinct global DNA methylation patterns.^8–10^ Classification of patients based on DNA methylation at five CpG sites has been shown to enable superior prediction of time to first treatment (TTT) and overall survival (OS) than *IGHV* status,^11^ demonstrating the potential clinical utility of epigenetic biomarkers.

While previously believed to be highly stable after diagnosis,^12^ recent studies have demonstrated evolution of the CLL epigenome over time.^7,13,14^ Greater plasticity of the CLL epigenome is associated with the acquisition of genetic aberrations such as del(17p),^13,15^ and worse patient outcomes such as shorter TTT.^13^ However, these studies have near-exclusively described global phenomena in the epigenome rather than identifying gene-specific changes, and therefore little is known about the acquired silencing or activation of genes implicated in response to therapy. In this study, we performed epigenome-wide analysis of DNA methylation to identify differentially methylated regions (DMRs) following exposure to therapy that may have utility as prognostic biomarkers and reveal genes with direct functional roles in chemosensitivity. We examined the association of the identified genes with patient prognosis in two independent cohorts and used cell-line and primary CLL cell models to study their functional impact on chemosensitivity. Our study revealed multiple DMRs that predict the duration of patient survival following therapy, and in particular identify *HOXA4* as an important regulator of sensitivity to multiple drugs used in the treatment of CLL.

## Methods

### Patient samples and sample preparation

An overview of the study approach and cohorts is provided in Supplementary Fig. 1. The study was primarily performed within a cohort (‘Newcastle cohort’) of 163 CLL patients attending the clinic at hospitals in the North–East of England (Freeman Hospital, Newcastle upon Tyne; Queen Elizabeth Hospital, Gateshead, and Sunderland Royal Hospital, Sunderland). The characteristics of the patients are provided in Supplementary Table 1. Data were collected on clinical characteristics and treatment history, and patient samples were analysed for *IGHV* mutational status, *CD38* expression and the presence of cytogenetic abnormalities (del(11q), del(13q), del(17p) and trisomy 12) and *TP53* and *ATM* mutations. Peripheral blood samples were taken from patients with white cell counts of >30  × 10^9^/L, from which mononuclear cells were isolated by density centrifugation using Lymphoprep media (Stem Cell Technologies) according to the manufacturer’s instructions. Genomic DNA was extracted from purified mononuclear cells using the Qiagen Blood and Tissue kit according to the manufacturer’s instructions.

### Epigenome-wide analysis of DNA methylation

The identification of leukaemia-specific DMRs was performed within the discovery cohort (*n* = 20), nested within the wider Newcastle cohort that was used for prognostic validation. Samples at multiple timepoints were available for a total of 42 of the 163 patients within the Newcastle cohort (median time between samples: 26.9 months). Samples were collected from patients presenting at the clinic with white cell counts of >30 × 10^9^/L and were selected to include patients who had received treatment between sampling, as well as those who remained treatment-naive. Of these 42 patients, 24 underwent treatment with fludarabine or chlorambucil between sampling, while 18 had stable disease and underwent no treatment. For DMR discovery, we utilised paired samples from 20 of the patients undergoing treatment and 4 who remained untreated. Clinical information for these patients is provided in Supplementary Table 2. In the absence of specific cell counts, B-cell composition of the samples was estimated by the Houseman method adapted by Horvath.^16,17^ The median B-cell composition was 95.4% (95% confidence interval (CI): 92.2–95.8).

Epigenome-wide analysis of DNA methylation at two timepoints was conducted using the Illumina HumanMethylation450 BeadChip platform, performed at the Edinburgh Clinical Research Facility, University of Edinburgh (United Kingdom), using 500 ng of DNA that was bisulfite-converted using the EZ DNA Methylation-Gold kit (Zymo Research) according to the manufacturer’s instructions. The data were processed in R using the Bioconductor package minfi, and differentially methylated regions were identified using the DMRcate package^18^ with *P* values adjusted for multiple-hypothesis testing by the Benjamini–Hochberg method. CLL-specific DMRs were identified by use of DNA methylation microarray data from matched purified samples of naive and class-switched memory B cells from the study of Kulis et al.,^10^ thereby enabling differentiation of CLL-specific methylation changes from those also seen during B-cell development.

### Validation cohort

DMRs identified in the discovery cohort were taken forward for examination in a validation cohort using publicly available Illumina HumanMethylation450 microarray data from the study of Tsagiopoulou et al.,^14^ available through ArrayExpress (E-MTAB-7575). Paired samples from 34 patients taken prior to treatment and at relapse were used to examine changes in methylation at the 31 DMRs by paired *t* test, with *P* values adjusted for multiple-hypothesis testing by the Benjamini–Hochberg method.

### Analysis of gene expression

The impact of epigenetic changes at CLL-specific DMRs was examined using Affymetrix Human Genome U133 Plus 2.0 gene expression microarray data from the study of Landau et al*.*,^5^ available through Gene Expression Omnibus (GSE37168). Data were leveraged from 13 patients for whom paired samples were taken prior to treatment and then at relapse following therapeutic intervention. Differential expression between timepoints was examined by paired *t* test, with *P* values adjusted for multiple-hypothesis testing by the Benjamini–Hochberg method. The correlation between DMR methylation and gene expression was assessed using paired DNA methylation and gene expression microarray data from a cohort of 139 CLL patients available through the International Cancer Genome Consortium (ICGC).

### Assessment of patient prognosis

The potential prognostic relevance of the validated DMRs was first examined using DNA methylation microarray data from 139 CLL patients within the ICGC cohort, for which samples were reported to comprise >95% neoplastic cells.^10^ Leading candidates were taken forward for further analysis within the Newcastle cohort (*n* = 163), following analysis of DMR methylation by pyrosequencing. Associations with post-treatment survival (i.e., time between first treatment and death or the last follow-up) were determined by Cox proportional hazard regression. Patients were stratified into high- and low-methylation categories for each DMR by ROC curve analysis using log2-transformed methylation values, with the optimal threshold determined by the Youden index (sensitivity + specificity−1). *P* values were adjusted for multiple-hypothesis testing by the Benjamini–Hochberg method.

### Pyrosequencing

Locus-specific analysis of *HOXA4* promoter methylation within the Newcastle cohort was performed by pyrosequencing. Samples from 163 patients were analysed (Supplementary Table 1), with sequential samples from 18 patients who remained treatment-naive at the time of the second sample used to confirm the specificity of DMR selection to therapeutic intervention. In total, 100 ng of DNA was bisulfite-modified using the Methylamp DNA modification kit (Epigentek) according to the manufacturer’s instructions, and the promoter region PCR-amplified using 1 µl of modified DNA. PCR products were prepared according to the manufacturer’s instructions and analysed on a PyroMark Q96 MD pyrosequencer (Biotage). The primer sequences used have been described previously.^19^ Analysis was performed in duplicate, with exclusion of samples where replicate mean values differed by >5%.

### Cell culture

The Raji cell line, a differentiated B-cell cell line derived from a patient with Burkitt’s lymphoma, was authenticated by STR profiling (NewGene, Newcastle upon Tyne, UK). Cells were cultured in RPMI 1640 media with 10% foetal calf serum. Primary CLL cells from patients within the Newcastle cohort were seeded onto a feeder layer of CD40L-expressing mouse fibroblast cells (a gift from Professor Chris Pepper, Brighton & Sussex Medical School) that were irradiated (30 Gy) to induce mitotic arrest, and then cultured in RPMI 1640 media with 10% foetal calf serum and 100 µg/ml IL4 (Sigma Aldrich).

### Lentiviral transduction

The impact of *HOXA4* expression upon drug sensitivity was analysed by lentiviral-based overexpression of the gene in Raji cells. This differentiated B-cell line was selected due to significantly superior transduction efficiency in comparison to the MEC1 chronic lymphocytic leukaemia cell line. Furthermore, Raji cells were more appropriate to examine drug response due to the relative insensitivity of MEC1 cells to clinically relevant doses of fludarabine due to a *TP53* mutation.^20,21^ Cells were transduced using 100 µl of lentivirus (pSINE-SIEW vector, a gift from Dr Paul Sinclair, Newcastle University Centre for Cancer, UK) that was concentrated 30-fold using Lenti-X solution (Clontech), and 8 mg/ml polybrene. Cells were washed in PBS after 24 h, and the efficiency of transduction measured by assessment of GFP expression by flow cytometry at day 5.

Primary CLL cells were transduced using 500 µl of lentivirus and 8 mg/ml polybrene, before seeding onto the feeder layer after incubation for 4 h. The efficiency of transduction was measured by flow cytometry-based analysis of GFP expression at day 5.

### Drug sensitivity

Transduced cells were treated with 1–50 µM of fludarabine (Sigma Aldrich), ibrutinib (Enzo Life Sciences) or idelalisib (Selleck Chemicals). Apoptosis was measured at 48 h using the Annexin V PE Apoptosis Detection Kit I (BD Biosciences) in conjunction with flow cytometry, using the BD FACSCanto II (BD Biosciences). Transduced primary CLL cells from three patients within the Newcastle cohort were grown on a CD40L-expressing feeder layer for 6 days prior to drug treatment. Experiments were performed in triplicate, and the results shown are the product of at least two separate experiments.

### Statistical analysis

Correlations between DMR methylation and gene expression were assessed by Spearman rank correlation. Associations of *HOXA4* methylation with cytogenetic abnormalities and *IGHV* status were identified by Fisher’s exact test, and correlations with *IGHV* sequence homology determined by Spearman rank correlation. Differences in *HOXA4* methylation by Binet stage were determined by Mann–Whitney *U* test, and associations with post treatment and overall survival were identified by Cox proportional hazard regression as previously described. Differential drug sensitivity and cell proliferation in transduced *HOXA4*-overexpressing cells were analysed by Mann–Whitney *U* test. All analyses were performed in R (version 3.2.5) and GraphPad Prism (GraphPad Software, version 7.0b). Statistical significance was defined as *P* < 0.05.

## Results

### Identification of differentially methylated regions following treatment

An outline of the study is shown in Supplementary Fig. 1. Firstly, to identify DMRs occurring in response to therapy, epigenome-wide analysis of DNA methylation was performed on 20 patients at two timepoints during treatment. The patients were nested within the Newcastle cohort of 163 patients attending the clinic in the North–East of England (Supplementary Table 1). The median time between sampling (timepoints ‘A’ and ‘B’) was 31.1 months. Further characteristics of the patients used for DMR discovery are provided in Supplementary Table 2.

Regional changes in methylation during the course of treatment were identified using the DMRcate approach.^18^ We restricted the output to DMRs with a maxbetafc (largest mean change in methylation, β, at a single CpG site) of >0.04 and with *P*_FDR_ < 0.005 within the region, which revealed 551 loci. We then selected for DMRs mapping to loci within 1500 bases of the transcriptional start site or within the 5′UTR or the first exon. A total of 490 DMRs were retained, each comprising 2–55 CpG sites, of which 433 were hypermethylated and 57 hypomethylated (Supplementary Table 3). Pyrosequencing-based validation of five DMRs confirmed the findings of the arrays (Supplementary Fig. 2).

### Leukaemia-specific acquisition of epigenetic traits

**Fig. 1:**
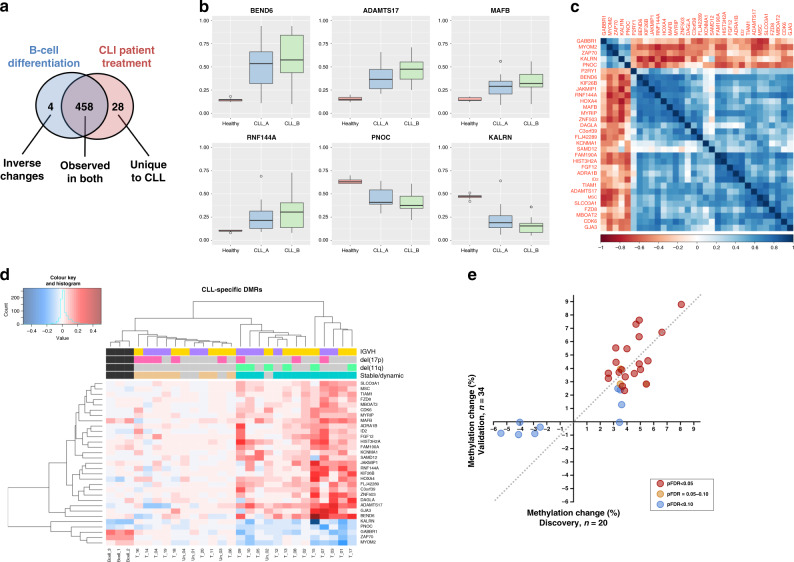
Characterisation of CLL-specific DMRs. **a** Venn diagram illustrating the similarities in methylation changes during B-cell differentiation and in CLL patients during the course of treatment. **b** Methylation at six representative DMRs in normal CD19 + B cells from 14 healthy individuals (‘Healthy’, red) and 20 CLL patients at timepoints A (‘CLL_A’, blue) and B (‘CLL_B’, green). **c** Correlation plot of changes in methylation (Δ*β*) at 32 CLL-specific DMRs. Squares are coloured by median correlation values (*r*), with positive correlations in blue and negative correlations in red. **d** Heatmap displaying changes in methylation at 31 treatment-specific DMRs in 20 CLL patients undergoing treatment (T_01–T_20) and 4 who remained untreated (Un_01–Un_04) between timepoints A and B, and changes in B-cell development from naive to memory class-switched (Bcell_1–Bcell_3). Increases in methylation are displayed in red, and decreases in blue. *IGHV*-unmutated cases are indicated in purple and *IGHV*-mutated cases in yellow, with 11q deletions (light green) and 17p deletions (pink) also indicated. Patients were classified as having stable (brown) or dynamic (turquoise) epigenetic patterns by hierarchical clustering. **e** Correlation in mean differential methylation of 31 DMRs observed in the discovery and validation cohorts. Circles represent DMRs and are shaded according to statistical significance as indicated, with a line of identity (dashed line).

It has recently been demonstrated that most methylation changes observed in CLL also occur during the later stages of B-cell differentiation.^13^ To reveal epigenetic changes more likely to be directly implicated in CLL pathobiology, we excluded regions that are differentially methylated during B-cell development. For this purpose, we utilised publicly available methylation microarray data from naive and memory B cells, obtained from the study of Kulis et al.^10^ The majority of identified changes occurring during treatment were mirrored by similar changes between naive and class-switched memory B cells (Fig. 1a). Our analysis revealed 32 DMRs that were putatively specific to CLL (Table 1 and Supplementary Fig. 3), being either unchanged (Δ*β* < 0.04) in B-cell development (28 DMRs) or displaying an inverse change (four DMRs). Of these, 27 displayed increased methylation and 5 reduced methylation in CLL following treatment (Fig. 1b). Changes in methylation were highly correlated between the DMRs (Fig. 1c), with a median absolute correlation (*r*) of 0.51. To further restrict our analysis to DMRs that are specifically selected for during treatment, we examined methylation of 32 DMRs in sequential samples from four patients who remained untreated between sampling. Only 1 of the 32 DMRs, which mapped to the *P2RY1* gene, displayed a significant change in methylation among untreated patients, and was subsequently excluded from further analysis.

**Table 1:** Differentially methylated regions in CLL following treatment.

Gene	DMR	Probes	Minpval	Max. *β* value change	Discovery			Validation			
					A	B	Mean diff	A	B	Mean diff	pFDR
*BEND6*	chr6:56819429–56819432	2	1.45 × 10^−4^	0.08	0.49	0.57	0.08	0.54	0.62	0.09	0.0482
*ADAMTS17*	chr15:100881458–100882165	4	5.53 × 10^−5^	0.10	0.40	0.47	0.07	0.49	0.56	0.07	0.0248
*HIST3H2A*	chr1:228645462–228645634	7	2.04 × 10^−9^	0.08	0.22	0.28	0.06	0.28	0.33	0.05	0.0248
*FGF12*	chr3:192127330–192127457	3	4.11 × 10^−6^	0.07	0.16	0.21	0.05	0.17	0.25	0.08	0.0248
*GJA3*	chr13:20735337–20735532	3	2.00 × 10^−4^	0.06	0.10	0.16	0.05	0.12	0.14	0.02	0.0562
*HOXA4*	chr7:27169957–27170554	8	2.41 × 10^−4^	0.10	0.60	0.65	0.05	0.59	0.63	0.04	0.0075
*JAKMIP1*	chr4:6201080–6202384	7	4.28 × 10^−6^	0.09	0.32	0.37	0.05	0.32	0.38	0.06	0.0482
*MAFB*	chr20:39317034–39318100	5	4.46 × 10^−9^	0.08	0.29	0.34	0.05	0.29	0.33	0.04	0.0301
*RNF144A*	chr2:7057153–7057945	7	1.61 × 10^−4^	0.07	0.24	0.29	0.05	0.32	0.40	0.07	0.0248
*ZNF503*	chr10:77161647–77161653	3	2.11 × 10^−7^	0.06	0.14	0.18	0.05	0.18	0.22	0.04	0.0482
*ADRA1B*	chr5:159343275–159343549	4	4.24 × 10^−4^	0.05	0.16	0.19	0.04	0.19	0.23	0.04	0.0248
*C3orf39*	chr3:43146909–43147587	6	1.50 × 10^−6^	0.06	0.25	0.28	0.04	0.27	0.30	0.03	0.0248
*CDK6*	chr7:92462202–92463218	5	2.41 × 10^−6^	0.05	0.13	0.17	0.04	0.23	0.25	0.02	0.0482
*DAGLA*	chr11:61447834–61447938	4	4.42 × 10^−4^	0.04	0.18	0.21	0.04	0.12	0.15	0.03	0.0248
*FAM190A*	chr4:91047852–91049533	11	3.83 × 10^−8^	0.08	0.19	0.22	0.04	0.20	0.23	0.03	0.0699
*FLJ42289*	chr15:100890243–100890462	6	1.33 × 10^−7^	0.06	0.19	0.23	0.04	0.26	0.31	0.05	0.0248
*ID2*	chr2:8822058–8822738	4	1.30 × 10^−6^	0.05	0.16	0.20	0.04	0.22	0.23	0.01	0.2915
*KCNMA1*	chr10:79397346–79398415	6	3.31 × 10^−6^	0.06	0.18	0.22	0.04	0.20	0.24	0.04	0.0848
*FZD8*	chr10:35929604–35931235	11	1.23 × 10^−4^	0.05	0.16	0.18	0.03	0.19	0.22	0.04	0.0482
*KIF26B*	chr1:245318209–245318335	5	5.95 × 10^−4^	0.06	0.09	0.12	0.03	0.14	0.14	0.00	0.9860
*MBOAT2*	chr2:9143747–9144505	7	3.14 × 10^−4^	0.04	0.17	0.19	0.03	0.19	0.22	0.03	0.0248
*MSC*	chr8:72754953–72757004	17	3.64 × 10^−9^	0.05	0.22	0.26	0.03	0.27	0.30	0.02	0.1298
*MYRIP*	chr3:39850280–39851931	12	2.43 × 10^−6^	0.06	0.17	0.21	0.03	0.19	0.23	0.04	0.0475
*SAMD12*	chr8:119634283–119634612	5	4.70 × 10^−4^	0.05	0.15	0.18	0.03	0.19	0.21	0.02	0.2383
*SLCO3A1*	chr15:92396240–92397541	9	1.31 × 10^−8^	0.11	0.12	0.15	0.03	0.15	0.20	0.06	0.0248
*TIAM1*	chr21:32931471–32931935	3	1.03 × 10^−4^	0.05	0.09	0.13	0.03	0.11	0.15	0.04	0.0345
*GABBR1*	chr6:29600994–29602034	7	1.98 × 10^−4^	−0.05	0.16	0.13	−0.03	0.12	0.11	−0.01	0.4471
*ZAP70*	chr2:98329337–98330020	7	1.66 × 10^−4^	−0.04	0.22	0.19	−0.03	0.17	0.17	0.00	0.4667
*MYOM2*	chr8:1993545–1993893	2	2.64 × 10^−4^	−0.05	0.33	0.29	−0.04	0.35	0.35	0.00	0.9860
*PNOC*	chr8:28173732–28174847	7	1.27 × 10^−7^	−0.07	0.43	0.39	−0.04	0.48	0.47	−0.01	0.4693
*KALRN*	chr3:124303035–124303404	4	3.34 × 10^−6^	−0.07	0.22	0.16	−0.05	0.15	0.15	−0.01	0.4072

The 31 CLL-specific DMRs are listed with a description of the genomic region (Human GRCh37/hg19 genomebuild), the number of CpG sites mapping to the DMR and the minimum p value (minpval) and largest mean change in methylation observed at individual CpG sites within the DMR (maxbetafc). The DMRs are ranked by the mean methylation change between timepoints (i.e., Δ*β*) in the discovery cohort, with the mean methylation levels (*β*) at the entry (A) and follow-up (B) timepoints displayed for both the discovery and validation cohorts.

While the mean changes in methylation (*β*) across all patients were moderate (−0.05–0.08, Table 1), a high degree of interpatient variability was observed at each DMR, as some patients displayed large alterations in methylation (max Δ*β*: 0.51), while others displayed no changes between the timepoints. This is exemplified by six patients displaying differential methylation of Δ*β* > 0.10 at 11–20 DMRs, while ten patients displayed such changes at only a single locus or not at all (Fig. 1d). Therefore, it is important to emphasise that epigenetic changes at these DMRs do not represent moderate alterations in methylation uniformly observed among all patients, but rather they are loci that are variably differentially methylated between individuals. Importantly, the magnitude of changes in methylation was not significantly associated with the time between sampling for any of the DMRs (Spearman’s rank correlation, *P*_FDR_: 0.25–0.82). Furthermore, patients who had previously been treatment-naive (*n* = 7) displayed no significant difference in response at any of the DMRs in comparison to those who had previously been treated (*n* = 13) (Mann–Whitney *U* test, *P*_FDR_ > 0.90).

Unsupervised hierarchical clustering identified 12 patients displaying changes in DNA methylation across 31 DMRs following therapy (‘Dynamic’), while 8 displayed highly stable methylation signatures (‘Stable’) (Fig. 1d). 11q deletions were exclusively observed among Dynamic patients (5 of 12) and never among 8 Stable patients (Fisher’s exact test, *P* = 0.055). No association was observed for the Dynamic group with either *IGHV* status (*P* = 1.00) or 17p deletions (*P* = 0.16). There was no enrichment among the groups for patients who were treatment-naive at timepoint A (Fisher’s exact test, *P* = 0.64).

### Examination of CLL-specific DMRs in the validation cohort

We performed validation of 31 CLL-specific DMRs using publicly available DNA methylation microarray data taken from 34 patients prior to first treatment and again at relapse.^14^ The median time between samples was 30 months, and patients were near-exclusively treated with FCR. There was a high degree of correlation in the mean methylation changes observed at 31 DMRs in the discovery and validation cohorts (*r* = 0.68, *P* < 0.0001) (Fig. 1e), with 19 showing significant differences in methylation between first treatment and relapse, and a further three approaching significance (*P*_FDR_ < 0.085) (Table 1).

### Differential expression of DMR-associated genes

**Fig. 2:**
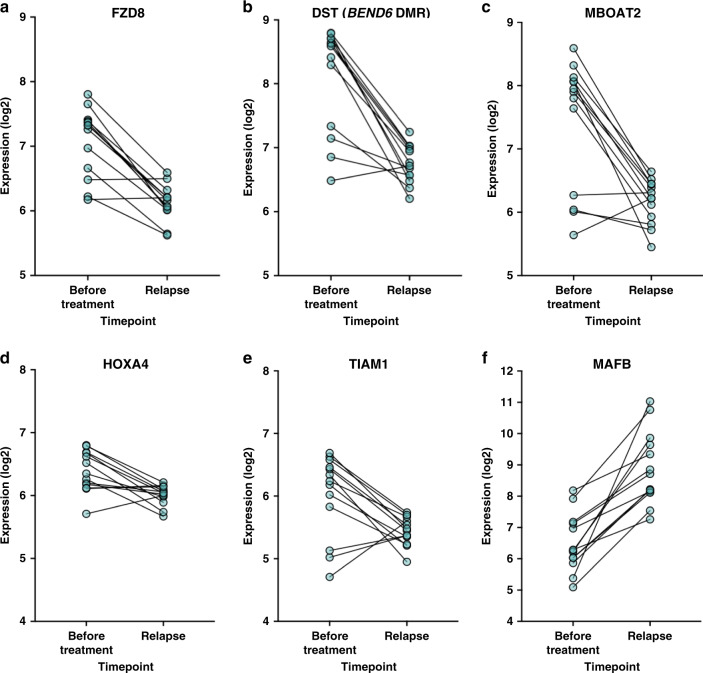
Differential expression of DMR-associated genes in the validation cohort. Analysis of the expression (log2 transformed) of *FZD8* (**a**), *DST* (*BEND6* DMR) (**b**), *MBOAT2* (**c**), *HOXA4* (**d**), *TIAM1* (**e**) and *MAFB* (**f**) in samples taken from 13 patients prior to chemotherapy and at relapse in the validation cohort (GSE37168). Lines indicate samples from the same patient.

To examine changes in the expression of DMR-associated genes following therapy, we utilised publicly available paired gene expression data from 13 patients taken before treatment and following chemotherapeutic intervention at the point of relapse (GSE37168). The median time between samples was 42 months, and the patients were primarily treated with fludarabine-based regimens.^5^ Of the 22 validated CLL-specific DMRs, 16 associated genes were differentially expressed between timepoints (*P*_FDR_ < 0.05), while a further gene, *RNF144A*, approached significance (*P*_FDR_ < 0.10). Twelve of these showed decreased expression (*ADAMTS17*, *CDK6*, *DAGLA*, *DST* (*BEND6* DMR), *FGF12*, *FZD8*, *HOXA4*, *KCNMA1*, *MBOAT2*, *MYRIP*, *RNF144A* and *TIAM1*, Fig. 2a–e and Supplementary Table 4) and five showed increased expression (*GJA3*, *JAKMIP1*, *MAFB*, *SLCO3A1* and *ZNF503*, Fig. 2f). Although promoter methylation is typically associated with inhibition of gene expression, approximately 30% of correlations between promoter methylation and gene expression are positive ones (as observed here).^22^ Interestingly, *CDK6* and *TIAM1* displayed simultaneous significant upregulation and downregulation of transcripts measured by different probes, suggesting an impact of chemotherapy upon transcript usage. Four genes displayed no significant change (*ADRA1B*, *CCSER1* (*FAM190A* DMR), *HIST3H2A* and *POMGNT2* (*C3orf39* DMR)), while *FLJ42289* could not be assessed due to no expression data being available.

Taken together, we identified 17 genes that demonstrate CLL-specific differential methylation and expression in patients following exposure to chemotherapy, as concurringly observed in three independent cohorts (details of the DMRs available within Supplementary Fig. 4). To further explore the functional basis of our observations, we assessed correlations between DMR methylation and gene expression using data from 139 CLL patients available through the ICGC. Five DMRs showed a significant correlation with gene expression (*CDK6*, *DST* (*BEND6* DMR) *DAGLA*, *SLCO3A1* and *ZNF503*, *P* < 0.05) and a further four approached significance (*ADAMTS17*, *HOXA4*, *MAFB* and *MYRIP*, *P* < 0.10). All exhibited negative correlations between DMR methylation and gene expression with the exception of *DAGLA*. It is known that CpG sites in close proximity to one another can exhibit conflicting associations with gene expression,^22^ which may explain discrepancies with our previous observations of changes in gene expression following chemotherapy. We noted reduced variation and lower levels of expression of the genes within the ICGC cohort in comparison to GSE37168, which may have impaired the ability to identify correlations at some other loci.

### Impact of DMR methylation upon post-treatment survival

**Fig. 3:**
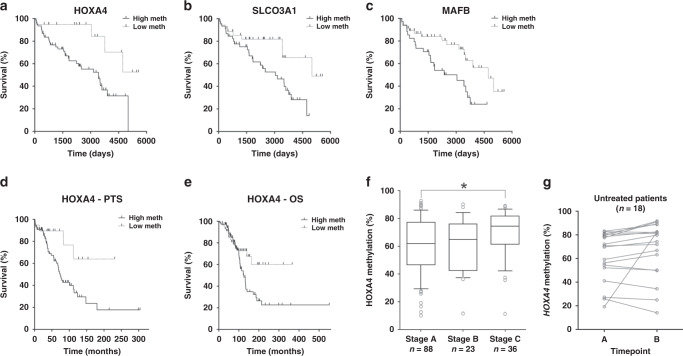
Associations between DMR methylation and patient prognosis. **a–c** Kaplan–Meier plots for post-treatment survival in patients within the ICGC cohort (*n* = 139) stratified by methylation level (high/low) of *HOXA4* (**a**), *SLCO3A1* (**b**) and *MAFB* (**c**). **d**–**g** Pyrosequencing-based analysis of *HOXA4* methylation within the Newcastle cohort (*n* = 163). Kaplan–Meier plots for post-treatment survival (**d**) and overall survival (**e**) by *HOXA4* methylation (high/low). *HOXA4* methylation by Binet stage (**f**), with lines indicating median values, boxes the interquartile range (IQR), whiskers the highest and lowest values within 1.5*IQR and outliers displayed as individual points. *HOXA4* methylation at sequential timepoints among 18 untreated patients (**g**), with lines indicating samples from the same patient.

The prognostic significance of the 9 DMRs with confirmed correlation between methylation and gene expression was examined in the same 139 CLL patients within the ICGC cohort.^10^ We examined methylation at each of the DMRs, here measured in early/pre-treatment samples, to determine association with post-treatment survival (i.e., the duration of survival after the initiation of therapy). We identified three DMRs that were significantly associated with patient post-treatment survival by univariate analysis: *HOXA4*, *MAFB* and *SLCO3A1* (Table 2 and Fig. 3a–c). Each displayed increased methylation in the discovery and validation cohorts, and hypermethylation was associated with reduced post-treatment survival in the ICGC cohort. A further three approached significance (*ADAMTS17*, *CDK6* and *MYRIP*). Therefore, our analyses identified three prognosis-associated DMRs that represent adverse epigenetic traits enriched during disease progression. The strongest effect on survival was with the *HOXA4* DMR, with hypermethylation associated with an ~3.5-fold increased risk of death following treatment.

**Table 2:** Associations of DMR methylation and patient post-treatment survival.

DMR	HR	95% CI	*P*_FDR_	
*HOXA4*	**3.48**	**1.71–7.08**	0.0445*	
*SLCO3A1*	**2.43**	**1.26–4.68**	0.0446*	
*MAFB*	**2.34**	**1.19–4.59**	0.0445*	
*ADAMTS17*	2.12	1.10–4.07	0.0607	
*ZNF503*	1.96	0.91–4.22	0.1660	
*MYRIP*	1.95	1.01–3.74	0.0820	
*CDK6*	1.88	0.96–3.66	0.0820	
*BEND6*	1.77	0.65–4.84	0.1660	
*DAGLA*	1.74	0.79–3.82	0.1390	

Univariate analysis of post-treatment survival in the ICGC cohort by methylation of the nine CLL-specific DMRs that were identified as differentially expressed following therapeutic intervention and displaying significant correlations between methylation and gene expression. The DMRs are ranked by hazard ratio (HR, with 95% confidence intervals), with significant results (*P*_FDR_ < 0.05) highlighted in bold and by an asterisk.

### Examination of prognosis-associated DMRs

We selected two of the prognosis-associated DMRs for further examination based on plausible biological roles in mediating patient response to therapy. *HOXA4* is a homeobox gene that encodes a transcription factor involved in development, and it has previously been reported as hypermethylated in CLL^23^ and is associated with imatinib resistance among chronic myeloid leukaemia patients.^24^ *SLCO3A1* encodes an anion transporter that may be implicated in drug uptake.

Examination of *SLCO3A1* DMR methylation in patient samples within the discovery cohort revealed a significant correlation with gene expression, but re-expression of the gene in the malignant B-cell lines NALM6 and SEM did not significantly impact upon their sensitivity to fludarabine (Supplementary Fig. 5), thereby suggesting that *SLCO3A1* re-expression in isolation has little impact on drug sensitivity.

### *HOXA4* hypermethylation and patient characteristics

To investigate the role of *HOXA4* in CLL and explore how it relates to the progression of the disease, we first analysed *HOXA4* promoter methylation by pyrosequencing in the Newcastle cohort (*n* = 163). Higher methylation levels were associated with reduced post-treatment survival (Fig. 3d) and overall survival (Fig. 3e) (*P* = 0.03 and *P* = 0.03, respectively), supporting the association with post-treatment survival previously observed in the ICGC cohort. We also identified a progressive increase in methylation with the progression of the disease (Fig. 3f). Median methylation levels increased from 62% among Binet Stage A patients to 65% at Stage B and 74% at Stage C, with the difference between Stage A and Stage C patients statistically significant (Mann–Whitney *U* test, *P* = 0.03). *HOXA4* hypermethylation was significantly associated with *IGHV* sequence homology (*r* = 0.34, *P* < 0.0001) and with 11q deletions (Fisher’s exact test, *P* = 0.001), but not 13q and 17p deletions (*P* = 0.63 and *P* = 0.79, respectively) or *CD38* expression (*r* = –0.005, *P* = 0.97) (Supplementary Table 5).

Among these 163 patients, sequential samples were available from 18 patients who underwent no treatment between the timepoints. Pyrosequencing-based analysis revealed *HOXA4* methylation to be stable among these untreated patients (Fig. 3g), in contrast to our previous observation of significantly increased methylation in response to treatment within the discovery cohort. These data provide further evidence that changes in *HOXA4* methylation are selected for patients following exposure to therapy.

### Re-expression of *HOXA4* increases sensitivity to multiple drugs used in CLL therapy

**Fig. 4:**
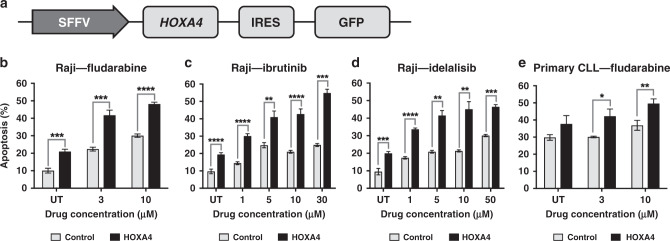
*HOXA4* expression is associated with increased drug sensitivity. **a** Diagrammatic representation of the lentiviral construct used to transduce Raji and primary CLL cells. The SFFV promoter, *HOXA4* gene, internal ribosomal entry site (IRES) and green fluorescent protein (GFP) gene are indicated. **b**–**d** Drug sensitivity of control (empty vector) and *HOXA4*-expressing transduced Raji cells in response to treatment with fludarabine (**b**), ibrutinib (**c**) and idelalisib (**d**). Apoptosis was measured 48 h after drug treatment by detection of Annexin V by flow cytometry. Mean values are displayed, with error bars indicating standard error of the mean. **e** Sensitivity of primary CLL cells transduced to express *HOXA4* to fludarabine treatment (*n* = 3). Apoptosis was measured 48 h after drug treatment by detection of Annexin V by flow cytometry. Mean values are displayed, with error bars corresponding to standard error of the mean. Statistical significance is indicated (*P < 0.05; **P < 0.01; ***P < 0.005; ****P < 0.001).

To determine whether *HOXA4* expression confers sensitivity to therapy, we used a lentiviral system to express *HOXA4* in Raji cells (Fig. 4a). Re-expression of *HOXA4* in transduced Raji cells was confirmed by qPCR (Supplementary Fig. 6). We observed significantly increased apoptosis (*p* < 0.05) in *HOXA4*-expressing cells 48 h after treatment with 3–10 µM fludarabine (Fig. 4b), 1–30 µM ibrutinib (Fig. 4c) and 1–50 µM idelalisib (Fig. 4d) in comparison to control cells transduced with an empty vector. Higher levels of apoptosis were also observed in untreated cells, but the increased sensitivity to drug exposure remained significant even after correction for this effect (Supplementary Fig. 7), indicating that re-expression increases sensitivity to fludarabine, ibrutinib and idelalisib.

To confirm that the observations in Raji cells were relevant to CLL, we transduced primary CLL cells from three patients with *HOXA4*-expressing and control lentiviral constructs. Primary cells were maintained on a feeder layer and then treated with 3 and 10 µM fludarabine. We observed significantly higher levels of apoptosis at both drug concentrations in primary CLL cells expressing HOXA4 in comparison to control cells (*P* = 0.02 and *P* < 0.01, respectively, Fig. 4e), confirming that re-expression of *HOXA4* increases drug sensitivity in primary CLL cells.

## Discussion

The chronic lymphocytic leukaemia epigenome was previously considered to be highly stable throughout the course of the disease,^12^ but it is increasingly recognised that it is dynamic.^13^ CLL is now understood to show selection for subclonal genetic aberrations with its progression,^5^ and our study has, for the first time, identified similar enrichment of abnormal gene-specific epigenetic traits that may be key to understanding patient response to therapy. Indeed, as it is still commonly perceived to be an incurable disease, elucidating the acquisition and effect of adverse genetic and epigenetic traits provides crucial insight into how the disease develops resistance to chemotherapy. Here, we have identified genes that are differentially methylated and expressed following exposure to therapy, and that are associated with post-treatment survival. In particular, we have identified enrichment for the epigenetic silencing of *HOXA4* that reduces the sensitivity of leukaemic cells to therapy and thereby impairs patient survival. To the best of our knowledge, ours is the first study to identify enrichment for locus-specific epigenetic traits following treatment that may predict patient outcome following initiation of therapy.

There is increasing evidence for evolution of the CLL epigenome during disease progression,^7,14^ with global trends in DNA methylation associated with the acquisition of genetic aberrations.^13,15^ These epigenetic changes are typically moderate, even when associated with progression from indolent disease to a more aggressive form requiring therapeutic intervention.^25^ Furthermore, many of these loci are also differentially methylated in B-cell development, with the CLL epigenome more closely resembling that of class-switched memory B cells than naive ones, regardless of *IGHV* status.^10,25^ Indeed, it is increasingly recognised that the overwhelming majority of the epigenetic changes observed in CLL are also seen during B-cell development.^8,13^ A recent examination of 31 genes previously reported to be hypermethylated in CLL across nine studies revealed that all but one gene, *HOXA4*, show similar hypermethylation during B-cell development.^8^ Consistent with this, we observed that most loci undergoing epigenetic changes during treatment were not unique to CLL. Unusually for terminally differentiated cells, B cells retain the ability to divide, and thus it may be that these methylation changes are reflective of B-cell proliferation, irrespective of the differentiation state or transformation. We therefore took steps to identify alterations unique to CLL in order to identify genes that may be directly implicated in response to therapy.

We have previously identified epigenetic dysregulation of *HOXA4* in both myeloid and lymphoid leukaemias,^23,26^ and reported that hypermethylation of *HOXA4* is associated with poor response to imatinib in chronic myeloid leukaemia.^23,24^ Here, we have built on these observations to reveal enrichment for *HOXA4* hypermethylation during the course of CLL patient treatment and disease progression, and to provide the first evidence that loss of *HOXA4* expression reduces the sensitivity of malignant B cells to multiple chemotherapeutic agents. Given the variable mechanisms of action of these drugs (fludarabine, ibrutinib and idelalisib), this suggests a broad anti-survival effect of the gene as opposed to a drug-specific one. This hypothesis is further supported by our observation of increased apoptosis in untreated cells expressing *HOXA4* in comparison to those that do not express it, implying activation of pro-survival signalling. Recent work in lung cancer cell lines has demonstrated that expression of *HOXA4* inhibits cell survival via inhibition of the Wnt signalling pathway,^27^ a pathway known to be activated in CLL,^28^ and which promotes the survival of leukaemic cells.^29^ Negative regulators of Wnt signalling are epigenetically silenced in CLL,^30^ and our study may implicate *HOXA4* as another silenced inhibitor. This is further supported by the observation of frequent hypermethylation of the *HOXA* cluster and hypomethylation of Wnt ligands in CLL patient samples.^31^ Subsequently, subclonal cell populations with biallelic methylation of *HOXA4* would be selected due to the increased pro-survival Wnt signalling, with selection pressure significantly increased with therapeutic intervention.

Previous studies have identified genetic aberrations present at low frequencies in early-stage CLL that already enables prediction of patient prognosis,^32,33^ and the expansion of such subclones following therapeutic intervention.^5^ Our study has similarly identified DNA methylation at three DMRs, including *HOXA4*, that when measured in early disease are able to predict post-treatment survival, consistent with the expansion of subclones with altered methylation that were already present prior to exposure to therapy. We have also provided further evidence to support the hypothesis that the epigenome displays co-evolution with genetic aberrations.^13^ Locally disordered methylation is enriched in patients displaying genetic evolution,^7^ and a recent study of 13 patients demonstrated expansion of genetic subclones only among those also displaying concordant changes in DNA methylation over time, irrespective of the *IGHV* subtype.^34^ Our results may suggest a potential association between patients with dynamic DNA methylation patterns and the acquisition of 11q deletions, an important marker of impaired survival and consistent disease progression.^35^ Our further observation of an association between *HOXA4* hypermethylation and 11q deletions is likely to be driven by this correlation with Dynamic cases, as it is unlikely that the two are causally linked. However, we were unable to confirm this association in the validation cohort (Fisher’s exact test, *P* = 0.71), and therefore it is not yet clear if this is a phenomenon in CLL or a cohort-specific observation. Further work is required to elucidate the possible relationship between dynamic epigenetic profiles and 11q deletions, especially as no significant association was observed with 17p deletions, thereby implying that there may be a more complex mechanism than general genomic instability. Interestingly, no association was present between *IGHV* mutational status and dynamic epigenetic profiles. This is in contrast to the work of Oakes et al.^13^ that suggested epigenomic heterogeneity and evolution are more common among higher-risk *IGHV*-unmutated cases, but supported by the observations of Smith et al.^25^ that differentially methylated loci in disease progression are not correlated with *IGHV* status.

The inherent variability between CLL patients in disease progression and treatment represents a significant challenge in its study. The heterogeneity in treatment types and histories among the patients used for DMR discovery represents a potential limitation in our analysis, but it has not prohibited the identification of DMRs following therapeutic intervention that were validated in independent cohorts. Furthermore, it ensures that the aberrant epigenetic traits identified in our study are of broad translational relevance and do not represent drug-specific effects, as supported by our in vitro findings of increased sensitivity to three chemotherapeutic agents with *HOXA4* expression. A particular strength of our study is the use of transduced cell lines and primary CLL cells to assess the effect of *HOXA4* overexpression upon drug sensitivity. Examination of patient samples simply categorised by *HOXA4* methylation level would be subject to the extensive interpatient heterogeneity for other factors that could influence drug response, but the use of a lentiviral system has enabled a more controlled and direct study of the effect of *HOXA4* expression.

Our study has for the first time provided evidence for enrichment of locus-specific epigenetic traits in CLL following therapeutic intervention that are associated with patient outcome. In particular, we have identified epigenetic silencing of *HOXA4* as implicated in reduced sensitivity to therapy. Further work is required to delineate patient response by treatment type and further elucidate the associations of these DMRs with patient prognosis.

## Supplementary information

Supplemental Material

## Data Availability

The Illumina Infinium HumanMethylation450 BeadChip microarray datasets from 24 patients at two timepoints during the course of treatment are available in the Gene Expression Omnibus repository (https://www.ncbi.nlm.nih.gov/geo/).
