# Supplementary material for: Epigenome-wide analysis reveals functional modulators of drug sensitivity and post-treatment survival in chronic lymphocytic leukaemia
Source: Br J Cancer. 2020 Oct 21;124(2):474–83. doi: 10.1038/s41416-020-01117-8 (PMC7852668; doi:10.1038/s41416-020-01117-8)
Supplement: Supplementary file 1 — Supplemental Material [file 41416_2020_1117_MOESM1_ESM.pdf]

Characteristic		*n*
Patients	Total	163
Gender	Male	103
	Female	60
Age	Median (range)	70 (32 - 92)
Binet stage	A	88
	B	23
	C	36
IGHV	Mutated	93
	Unmutated	64
CD38	Low (<30%)	57
	High (>30%)	24
Cytogenetics	del(11q)	23
	del(13q)	91
	del(17p)	15
	Trisomy12	17
Mutations	*ATM*	8 / 70 (11.4%)
	TP53	21 / 160 (13.1%)
Follow-up	Median (range)	90 (4 – 551)

Supplementary Table 1: Characteristics of CLL patients within the Newcastle cohort. The age (years), gender, Binet stage at first sampling, *IGHV* mutation status, *CD38* expression, presence of cytogenetic abnormalities and mutations in *ATM* and *TP53*, and length of follow-up (months) are provided. Data on Binet stage was not available for 16 patients.

Patient	Age	Sex	Stage	*IGHV*	Cytogenetics	Time	Naive	CLB	F	C	R	Other
CLL_1	66	M	A	M	None	10		✓		✓		Az
CLL_2	68	M	C	M	13q	90			✓	✓	✓	Be
CLL_3	58	M	C	U	13q	65		✓	✓	✓	✓	Hy, On
CLL_4	59	M	C	U	17p	9	✓	✓	✓	✓		Ca
CLL_8	66	M	A	U	Tri12	56	✓	✓	✓	✓		
CLL_9	80	F	C	M	13q	50			✓	✓		
CLL_10	38	M	B	U	13q	88	✓	✓	✓	✓	✓	
CLL_11	60	F	B	M	13q	111		✓	✓	✓	✓	
CLL_12	60	F	B	U	11q, 13q, 17p	8			✓	✓		
CLL_13	60	M	C	U	11q, 13q	79		✓	✓	✓	✓	Ca, BMT, Be, Ib
CLL_14	72	F	A	M	13q	23		✓				
CLL_15	61	F	-	U	13q	16	✓	✓				
CLL_16	67	M	A	M	11q, 13q	41	✓		✓	✓	✓	Be
CLL_18	70	F	C	U	13q, 17p	26	✓			✓		
CLL_19	62	M	A	M	11q, 13q	29		✓				
CLL_20	60	M	A	M	13q, 17p	33			✓	✓	✓	
CLL_21	71	F	A	M	11q, 13q, Tri12	54	✓	✓			✓	Ib
CLL_22	57	M	B	M	13q, 17p	23			✓	✓	✓	
CLL_23	78	F	A	U	13q	12		✓			✓	Id
CLL_24	79	M	B	U	Tri12	1		✓				

Supplementary Table 2: Characteristics of the patients used in DMR discovery

The age at first sample, gender, Binet stage (A-C), *IGHV* mutation status, cytogenetic abnormalities (del(11q), del(13q), del(17p), and trisomy 12) and time between samples (months) are provided for the 20 CLL patients. Patients who were treatment naïve at Time-point A are indicated. Treatment between the two samplings are described (CLB = chlorambucil; F = fludarabine; C = cyclophosphamide; R = rituximab; Az = azathioprine; Hy = hydroxydaunorubicin; On = oncovin; Ca = campath; BMT = allogenic bone marrow transplant; Be = bendamustine; Ib = ibrutinib; Id = idelalisib).

gene_assoc	group	hg19coord	meanpval	maxbetafc	no.probes	minpval
AADAT	Body,5'UTR,1stExon,TSS200,TSS1500	chr4:171010728-171011615	1.62E-05	0.0640398	11	3.10E-06
ACCN1	1stExon,5'UTR,TSS200,TSS1500	chr17:32483560-32484259	9.05E-07	0.0876677	13	2.36E-07
ACY3	5'UTR,1stExon,TSS200,TSS1500	chr11:67417958-67418405	0.0001	-0.0543175	13	4.63E-05
ADAM12	Body,1stExon,5'UTR,TSS200,TSS1500	chr10:128076260-128077376	0.0005228	0.0506308	11	6.57E-06
ADAM32	TSS1500,TSS200,1stExon,5'UTR,Body	chr8:38964500-38965492	0.000805	0.0678658	12	0.0003147
ADAMTS16	TSS1500	chr5:5139069-5140029	5.05E-06	0.0523046	12	6.32E-10
ADAMTS17	Body,5'UTR,1stExon,TSS200,TSS1500	chr15:100881458-100882647	0.0004067	0.0968022	11	5.53E-05
ADAMTS18	Body,1stExon,5'UTR,TSS200	chr16:77467409-77469167	0.0020949	0.083277	8	0.0002937
ADAMTS19	TSS1500,TSS200,1stExon,Body	chr5:128795472-128797013	0.0010612	0.0502989	12	2.32E-05
ADAT3,SCAMP4	5'UTR	chr19:1907677-1908388	0.0001277	-0.0769294	5	5.17E-05
ADCY8	5'UTR,1stExon,TSS200,TSS1500	chr8:132052702-132054555	0.0013899	0.0552179	12	0.000171
ADRA1A	1stExon,5'UTR,TSS1500	chr8:26721870-26724234	0.0038021	0.0513495	7	0.0002946
ADRA1B	TSS1500,TSS200,5'UTR,1stExon	chr5:159343148-159343871	0.0009796	0.0539639	8	0.0004243
ADRA1D	1stExon,5'UTR,TSS1500	chr20:4229561-4230822	0.0006256	0.0851972	6	6.15E-06
ADRA2C	TSS1500,TSS200,1stExon	chr4:3768078-3768638	5.18E-09	0.0833729	6	4.46E-12
AFAP1	5'UTR,1stExon,TSS200,TSS1500	chr4:7941431-7942031	0.0006589	0.0625794	7	3.78E-05
AFAP1L1	TSS1500,TSS200,Body	chr5:148650899-148652108	8.06E-05	0.0615325	8	8.53E-09
AFF3	5'UTR,1stExon,TSS200,TSS1500	chr2:100721844-100723240	0.0016388	-0.075279	8	0.000295
AGAP1	TSS200,1stExon,5'UTR,Body	chr2:236402544-236404028	0.000905	0.0727921	7	1.20E-05
AGBL4	Body,1stExon,5'UTR,TSS200,TSS1500	chr1:50489240-50489954	1.02E-08	0.0722966	15	5.37E-09
AHNAK	TSS200,TSS1500	chr11:62314417-62315320	0.0015209	-0.0801008	7	0.0001534
AJAP1	TSS1500,TSS200,5'UTR,1stExon	chr1:4714008-4715467	0.0016185	0.0754332	8	2.03E-06
ALDH1A2	Body,5'UTR,1stExon,TSS200,TSS1500	chr15:58357204-58358384	0.0003366	0.1046758	10	1.90E-05
ALDH1L1	5'UTR,1stExon,TSS200,TSS1500	chr3:125898680-125899925	0.0023294	0.0511756	14	0.000145
ALDH8A1	1stExon,5'UTR,TSS200,TSS1500	chr6:135271056-135271801	0.0018047	-0.0701047	6	0.0003566
ALX1	TSS1500,TSS200	chr12:85673200-85673860	0.0022262	0.080172	5	0.0003788
ALX4	1stExon,5'UTR,TSS200,TSS1500	chr11:44331362-44333192	0.0002582	0.0741744	44	4.57E-12
ARHGAP12	5'UTR	chr10:32216031-32216390	0.0003329	-0.0742398	6	0.0001636
ARHGAP29	5'UTR,1stExon,TSS200,TSS1500	chr1:94702435-94704178	1.60E-06	0.108141	15	1.50E-13
ARMC3	TSS1500,TSS200,5'UTR,1stExon	chr10:23216637-23217173	0.0001357	0.073207	8	9.40E-05
ASCL1	5'UTR,1stExon	chr12:103351987-103352694	0.000286	0.0615579	8	0.0001172
ASCL2	1stExon,5'UTR,TSS200,TSS1500	chr11:2291260-2292787	0.0006509	0.063987	22	3.95E-06
ASPG	TSS1500,TSS200,Body	chr14:104551553-104552397	2.52E-05	0.081423	7	5.13E-06
ASXL3	TSS1500,TSS200,5'UTR,1stExon,Body	chr18:31158105-31158831	0.0005572	0.0505634	7	0.0002601
ATOH1	TSS1500,TSS200,1stExon	chr4:94749496-94750704	0.0009323	0.0685951	11	0.0002091
ATP10A	Body,1stExon,5'UTR,TSS200,TSS1500	chr15:26107658-26108948	6.55E-05	0.0645686	20	5.31E-08
AURKC	TSS1500,TSS200,5'UTR,1stExon	chr19:57741988-57742444	0.0001457	0.0442996	9	7.67E-05
B3GALT4,WDR46	1stExon,5'UTR,3'UTR,Body	chr6:33244976-33247509	0.0001551	-0.0542711	55	6.15E-08
BARHL2	1stExon,5'UTR,TSS200,TSS1500	chr1:91182128-91185233	0.0010326	0.1022873	22	1.73E-09
BAT1	Body,5'UTR	chr6:31507316-31508665	0.0005714	-0.0467716	12	6.86E-05
BAT2,SNORA38	5'UTR,TSS1500	chr6:31589161-31589994	0.0010849	-0.0504519	8	3.80E-05
BCL11B	Body,1stExon,5'UTR,TSS200,TSS1500	chr14:99736143-99738493	0.0019123	0.0689774	14	1.89E-05
BDNF	Body,5'UTR,1stExon	chr11:27740161-27741077	0.0006752	0.0910943	5	1.50E-09
BDNF	TSS1500	chr11:27744049-27744759	4.70E-05	0.0434618	6	4.90E-06
BEND6,DST	TSS1500,1stExon,TSS200,5'UTR	chr6:56819298-56820386	0.000248	0.0843854	9	1.72E-05
BMPR1B	TSS1500,TSS200,5'UTR	chr4:95678703-95679808	0.0015686	0.0659536	8	2.73E-05
BOK	TSS1500,TSS200,5'UTR,1stExon	chr2:242497595-242498649	0.0012604	0.0624005	7	0.0002113
BRUNOL4	1stExon,5'UTR,TSS200,TSS1500	chr18:35145353-35147826	8.27E-05	0.0602628	34	1.86E-11
C10orf107	TSS1500,TSS200,1stExon,5'UTR	chr10:63422254-63422841	8.49E-06	0.075004	9	1.90E-06
C10orf53	TSS1500,TSS200,5'UTR,1stExon,Body	chr10:50886878-50887934	9.82E-05	0.055215	13	2.11E-08
C10orf58	TSS1500,TSS200,1stExon,5'UTR	chr10:82167757-82168295	0.0007922	0.0432999	8	0.0003948
C12orf56	Body,1stExon,TSS200,TSS1500	chr12:64783703-64784686	4.62E-06	0.0660743	10	3.28E-07
C1orf115	TSS1500,TSS200,1stExon,Body	chr1:220863263-220864321	0.001676	0.0706106	11	3.23E-06
C1QTNF7	TSS1500,Body,TSS200,1stExon,5'UTR	chr4:15374950-15376180	0.0048457	-0.0777932	10	6.46E-05
C3orf39	5'UTR,1stExon,TSS200,TSS1500	chr3:43146780-43148002	9.46E-05	0.0556891	14	1.50E-06
C4orf19	TSS1500,TSS200,5'UTR	chr4:37455280-37455756	6.42E-06	0.0829016	9	5.44E-06
C6orf155	Body,TSS200,TSS1500	chr6:72130209-72131020	1.52E-06	0.0844763	12	9.93E-07
C6orf47	1stExon,5'UTR	chr6:31626915-31628440	0.0007851	-0.0632386	28	7.67E-12
CACNA2D1	Body,5'UTR,1stExon,TSS1500	chr7:82072332-82073810	0.002971	0.0480425	10	0.0002584
CACNG2	TSS200,TSS1500	chr22:37099037-37099696	0.0030087	0.0573311	7	0.0001114
CACNG4	TSS1500,1stExon,Body	chr17:64960668-64961914	0.0012068	0.0576186	8	0.0001535
CADM1	Body,1stExon,5'UTR,TSS200,TSS1500	chr11:115373757-115376182	0.0006399	0.0570639	17	1.88E-06
CALHM1	1stExon,5'UTR,TSS200,TSS1500	chr10:105218286-105219068	0.0015468	-0.0629151	7	0.0002804
CASR	TSS200,5'UTR,1stExon	chr3:121902442-121903095	8.11E-06	0.0463501	9	2.05E-07
CBLN1	1stExon,5'UTR,TSS200,TSS1500	chr16:49315302-49316883	0.0002768	0.0871856	8	8.52E-10
CCDC67	TSS1500,TSS200,1stExon,5'UTR	chr11:93063516-93064234	0.0013064	0.0898804	7	0.0004876
CCHCR1,TCF19	TSS1500,5'UTR,Body	chr6:31126689-31127863	0.0004144	-0.0457948	19	3.01E-07
CD300LB	1stExon,5'UTR,TSS1500	chr17:72527515-72528764	0.0041342	0.0522423	5	0.0003382
CDH2	Body,1stExon,5'UTR,TSS200,TSS1500	chr18:25755608-25758159	0.0014971	0.0807173	13	3.39E-07
CDH4	TSS1500,Body	chr20:59827195-59829060	0.0009909	0.1005206	6	0.0001326
CDK6	Body,5'UTR,1stExon,TSS200,TSS1500	chr7:92462202-92465342	0.0019192	0.052662	22	2.41E-06
CELSR3	1stExon	chr3:48698519-48699637	0.0018921	0.0533766	6	0.0002704
CHRDL1	5'UTR,1stExon,TSS200,TSS1500	chrX:110038886-110039604	2.14E-05	0.0525686	16	1.26E-05
CHST8	TSS1500,TSS200,1stExon,5'UTR	chr19:34112111-34114048	0.0007805	0.0865642	15	1.79E-11
CLDN10	TSS200,Body,5'UTR,1stExon	chr13:96204854-96205960	0.0031322	0.0755765	12	0.0002488
CLDN9	TSS200,1stExon,5'UTR	chr16:3062349-3062975	0.0015734	-0.0541507	8	3.88E-05
CLEC14A	1stExon,5'UTR	chr14:38724255-38725536	0.0017877	0.0813014	5	2.35E-05
CNTNAP2	TSS1500,TSS200,1stExon,5'UTR,Body	chr7:145812842-145814306	2.71E-06	0.0716252	17	1.38E-14
COBL	Body,1stExon,5'UTR,TSS200,TSS1500	chr7:51383119-51384937	0.0003277	0.0794838	13	4.07E-10
COL12A1	5'UTR,TSS200,TSS1500	chr6:75915250-75916445	0.0003428	0.0531772	13	4.15E-05
COL25A1	Body,5'UTR,1stExon,TSS200,TSS1500	chr4:110223040-110224951	2.20E-05	0.1209085	20	1.65E-12
CPT1B,CHKB-CPT1B,CHKB	5'UTR,Body,1stExon,TSS200,TSS1500,3'UTR	chr22:51016501-51017432	8.61E-05	0.0479213	14	3.83E-08
CRMP1	1stExon,TSS200,TSS1500	chr4:5894691-5895410	0.0001076	0.0852632	5	3.60E-06
CSDC2	TSS1500,TSS200,5'UTR	chr22:41956440-41957443	0.0004729	-0.0456271	6	1.63E-05
CT62	5'UTR,1stExon,TSS1500	chr15:71407484-71408914	0.0022241	0.0629924	11	7.11E-05
CTNND2	Body,5'UTR,1stExon,TSS200,TSS1500	chr5:11903145-11904912	7.35E-05	0.0482169	14	5.96E-12

CTTNBP2	Body,TSS1500	chr7:117512803-117513956	0.0026986	0.0499913	8	9.69E-05
CUGBP2	Body,TSS1500,TSS200,5'UTR,1stExon	chr10:11059290-11060652	0.0002897	0.0655306	14	4.54E-05
CXCL12	Body,TSS200,TSS1500	chr10:44880128-44881551	0.0007457	0.0687279	10	8.09E-06
CXCL2	Body,1stExon,5'UTR,TSS200,TSS1500	chr4:74964260-74965755	3.66E-05	0.0867619	11	1.56E-08
CYP2S1	TSS1500,TSS200,1stExon,Body	chr19:41698548-41699364	0.0003347	0.0576143	12	0.0002485
DAGLA	TSS1500,TSS200,5'UTR,1stExon	chr11:61447487-61448170	0.0010248	0.043585	7	0.0004082
DBC1	5'UTR,1stExon,TSS200,TSS1500	chr9:122131497-122132379	0.0010097	0.0657517	8	0.0004163
DBX1	Body,1stExon,TSS200,TSS1500	chr11:20180336-20182324	0.0007348	0.0807206	13	1.89E-07
DDIT4L	5'UTR,TSS200,TSS1500	chr4:101111147-101112317	0.0010656	0.0554771	10	0.0002162
DECR2,NME4	TSS1500,3'UTR	chr16:450562-451040	0.0005837	-0.0538006	5	0.000377
DIO3,MIR1247	TSS1500,TSS200,5'UTR,1stExon	chr14:102025815-102027797	1.39E-08	0.0774251	21	9.39E-16
DKK1	TSS1500,TSS200,5'UTR,1stExon,Body	chr10:54073548-54075082	0.0011815	0.0726861	12	1.31E-06
DKK3	Body,1stExon,TSS200,5'UTR,TSS1500	chr11:12029738-12031314	0.0002238	0.0783015	20	7.02E-08
DLC1	Body,1stExon,5'UTR,TSS200,TSS1500	chr8:12990213-12991196	0.0009319	0.0648313	11	0.000316
DLK1	TSS1500,1stExon,5'UTR	chr14:101192591-101193432	0.0012118	0.0785403	12	4.70E-08
DLX1	TSS1500,TSS200,5'UTR,1stExon,Body	chr2:172948825-172952415	0.0019243	0.0766714	24	9.99E-06
DMRTA2	5'UTR,1stExon,TSS200,TSS1500	chr1:50887944-50889510	0.0011945	0.0898301	12	1.56E-06
DOC2B	Body,TSS200,TSS1500	chr17:30635-31867	0.0010974	0.0459644	5	0.0004181
DOCK1	TSS1500,TSS200,Body	chr10:128593624-128594417	2.62E-08	0.1328454	8	3.71E-09
DOCK1,FAM196A	Body,TSS200,TSS1500	chr10:128994432-128995192	0.0021488	0.0570185	8	6.06E-05
DOCK5	TSS1500,1stExon,5'UTR,Body	chr8:25041357-25042474	0.0014251	0.0657872	7	2.14E-05
DPP10	TSS1500,Body,1stExon,5'UTR	chr2:115918745-115920474	0.0002209	0.1207182	9	2.39E-08
DPP6	TSS1500,TSS200,1stExon,5'UTR,Body	chr7:153583563-153585368	0.0004492	0.0749058	10	1.65E-06
DRD1	TSS200,TSS1500	chr5:174871289-174872330	0.0015841	0.0502729	6	0.0002119
DSC3	Body,1stExon,5'UTR,TSS200,TSS1500	chr18:28621490-28623874	0.0005606	0.0957952	17	1.35E-08
EBF2	Body,1stExon,TSS200,TSS1500	chr8:25898839-25902980	0.0007386	0.0774367	18	1.71E-08
EBF3	Body,5'UTR,1stExon,TSS1500	chr10:131761150-131763929	0.0003017	0.0874646	17	3.68E-08
EDNRB	Body,1stExon,5'UTR,TSS200,TSS1500	chr13:78491982-78494067	0.0002804	0.0967749	44	1.70E-08
EFCAB1	Body,1stExon,5'UTR,TSS200,TSS1500	chr8:49647579-49648363	9.83E-05	0.0462399	11	3.34E-05
EGLN3	Body,1stExon,5'UTR,TSS200,TSS1500	chr14:34419316-34420683	1.58E-05	0.0552709	14	1.63E-07
ELAVL3	Body,5'UTR,1stExon,TSS200,TSS1500	chr19:11591185-11592200	0.0014324	0.0633806	7	7.35E-05
ELAVL4	TSS1500,TSS200,1stExon,5'UTR,Body	chr1:50513166-50514460	3.73E-05	0.0907065	10	1.84E-07
EMX2,EMX2OS	TSS1500,Body,TSS200,1stExon,5'UTR	chr10:119300942-119305095	0.0018448	0.0639927	27	9.57E-07
EN1	1stExon,5'UTR	chr2:119604270-119605441	0.0027809	0.0524495	8	0.000498
EN2	TSS1500,1stExon,5'UTR	chr7:155249398-155251040	0.0003369	0.0821064	12	4.74E-05
EOMES	Body,1stExon,TSS200,TSS1500	chr3:27762636-27766054	0.0014537	0.064191	19	1.08E-08
EPHA6	TSS1500,TSS200,1stExon,Body	chr3:96532640-96534005	0.0004974	0.0731596	12	2.02E-06
EPO	TSS1500,TSS200,Body	chr7:100317741-100318718	0.0008012	0.0423733	11	0.0004586
ESX1	Body,5'UTR,1stExon,TSS200,TSS1500	chrX:103498900-103500137	0.0002724	0.0873682	16	6.83E-06
ESYT3	TSS1500,TSS200,1stExon,5'UTR	chr3:138152902-138153832	0.0015695	0.0749231	12	0.0004016
EVX2	Body,1stExon,5'UTR,TSS200,TSS1500	chr2:176947786-176950736	0.0011097	0.0700193	20	2.89E-06
EYA4	TSS1500,TSS200,5'UTR,1stExon	chr6:133561368-133563382	4.37E-05	0.0675662	43	1.57E-14
FAM110B	5'UTR	chr8:59057462-59058660	0.0013382	0.0645986	6	1.02E-05
FAM129A	Body,1stExon,5'UTR,TSS1500	chr1:184943055-184944288	0.0010145	-0.0519752	10	2.71E-05
FAM155A	1stExon,5'UTR,TSS200,TSS1500	chr13:108518955-108520945	0.001231	0.0471683	15	3.63E-07
FAM155B	TSS1500,TSS200,1stExon,5'UTR	chrX:68724275-68725815	0.0009449	0.0848615	9	1.54E-07
FAM190A	TSS1500,TSS200,5'UTR,1stExon	chr4:91047852-91049533	0.0007255	0.0765752	11	3.83E-08
FAM19A5	Body,TSS1500,TSS200,1stExon	chr22:48970993-48972349	0.0035744	0.0447552	5	0.000108
FAM38B	Body,5'UTR,1stExon,TSS200,TSS1500	chr18:11148510-11149841	0.0008714	0.0870999	13	2.33E-05
FAM84A	TSS1500,TSS200,1stExon,5'UTR,Body	chr2:14772312-14774902	0.0020416	0.0761081	18	1.97E-05
FAM84B	Body,5'UTR,1stExon,TSS200,TSS1500	chr8:127569163-127570908	5.86E-05	0.0496379	11	2.50E-08
FAT1	5'UTR,TSS200,TSS1500	chr4:187644620-187647197	0.0002649	0.0958532	18	2.69E-13
FBLL1	TSS1500,TSS200,Body	chr5:167955758-167957361	0.0002924	0.075382	10	6.50E-06
FBN2	Body,1stExon,5'UTR,TSS1500	chr5:127872958-127874825	0.0004348	0.062857	11	2.94E-05
FEZF1	Body,1stExon,5'UTR,TSS200,TSS1500	chr7:121943990-121945743	0.0002999	0.0724333	16	3.28E-07
FEZF2	Body,5'UTR	chr3:62357769-62358825	0.0008048	0.0485165	6	0.0001977
FGF12	TSS200,Body,TSS1500	chr3:192126935-192127991	0.0009634	0.0695044	8	4.11E-06
FGF3	Body,1stExon,5'UTR,TSS200,TSS1500	chr11:69631981-69634648	0.0020554	0.0464358	23	6.14E-05
FGFR2	5'UTR,TSS1500,1stExon,TSS200	chr10:123357075-123358317	0.0004891	0.0561029	9	1.65E-06
FLJ42289	Body,TSS200,TSS1500	chr15:100889683-100890996	0.000128	0.0558801	14	1.33E-07
FLJ45983,GATA3	Body,TSS1500,TSS200,5'UTR,1stExon	chr10:8094431-8098328	0.000293	0.0678419	55	1.41E-16
FLRT2	TSS1500,TSS200,1stExon,5'UTR	chr14:85995859-85998669	0.0005945	0.0842342	20	3.63E-09
FOXA1	Body,1stExon,5'UTR,TSS200,TSS1500	chr14:38063813-38065435	0.0005668	0.0683904	13	4.64E-09
FOXA2	5'UTR,1stExon,TSS200,TSS1500	chr20:22564947-22566289	0.0021594	0.0837245	8	2.99E-05
FOXB1	TSS1500,TSS200,5'UTR,1stExon	chr15:60294120-60296996	4.25E-05	0.1160104	18	2.42E-07
FOXB2	TSS1500,TSS200	chr9:79633350-79634509	0.0004892	0.0873946	5	0.0001122
FOXC2	TSS1500	chr16:86599714-86600499	0.0013457	0.1075742	6	2.66E-05
FOXD3	TSS1500,TSS200,1stExon	chr1:63787586-63789500	0.0016993	0.073596	10	0.0001912
FOXE3	1stExon,3'UTR	chr1:47882265-47883234	0.0003685	0.0930588	7	2.66E-05
FOXF2	TSS200,1stExon	chr6:1389966-1391207	0.0021304	0.0785181	5	5.43E-06
FOXG1	TSS1500,TSS200,1stExon,5'UTR	chr14:29234978-29236898	0.0013013	0.076718	23	1.35E-07
FOXI2	TSS1500,TSS200,1stExon	chr10:129535138-129535898	4.74E-06	0.0828132	10	9.49E-07
FOXI3	Body,1stExon,TSS200,TSS1500	chr2:88751405-88752836	0.000912	0.0586444	11	5.39E-05
FOXQ1	TSS1500,TSS200,5'UTR,1stExon	chr6:1311825-1313730	0.0002222	0.0567301	12	1.20E-08
FRK	1stExon,5'UTR,TSS200,TSS1500	chr6:116381609-116382179	0.0001425	-0.0620764	6	0.0001202
FSTL4	5'UTR,1stExon,TSS200,TSS1500	chr5:132947278-132948515	0.0012436	0.0859543	9	1.58E-05
FZD10	TSS1500,TSS200,5'UTR,1stExon	chr12:130646129-130647289	0.0017043	0.0426028	8	2.61E-06
FZD8	1stExon,TSS200,TSS1500	chr10:35929604-35931235	0.0016609	0.0495675	11	0.0001227
GABBR1	TSS200,TSS1500	chr6:29600994-29602034	0.0016345	-0.0463	7	0.000198
GABRE	Body,1stExon,5'UTR,TSS200,TSS1500	chrX:151142772-151143686	0.0003149	0.0814669	8	5.15E-06
GABRG3	TSS1500,TSS200,5'UTR,1stExon,Body	chr15:27214606-27217186	0.0010179	0.0810883	19	4.14E-07
GALNTL6	TSS1500,TSS200,1stExon,5'UTR	chr4:172733566-172734843	0.0009179	0.0614631	12	2.29E-05
GALR1	TSS1500,TSS200,1stExon,5'UTR	chr18:74960629-74962794	2.59E-07	0.0669633	30	4.05E-18
GAS2L3	TSS1500,TSS200,5'UTR,1stExon	chr12:100967130-100967923	4.73E-06	0.066502	9	2.44E-06
GATA4	TSS1500,TSS200,1stExon,5'UTR	chr8:11560540-11562367	0.0010935	0.0534202	20	3.60E-07
GATA5	Body,5'UTR,1stExon,TSS200,TSS1500	chr20:61050560-61051667	0.0010971	0.0418217	18	1.71E-05
GBX2	Body,1stExon,TSS200,TSS1500	chr2:237075576-237078223	0.0002078	0.1088716	16	4.83E-11

GDF6	1stExon,5'UTR,TSS200,TSS1500	chr8:97172825-97173722	0.0017948	0.0560447	7	0.0002932
GDNF	5'UTR,1stExon,TSS200,TSS1500	chr5:37837978-37840839	0.0016991	0.0806993	19	2.45E-08
GFRA1	Body,5'UTR,1stExon	chr10:118030848-118031654	0.0019547	0.0457605	6	0.0002369
GHSR	1stExon,TSS200,TSS1500	chr3:172165517-172166920	0.0021911	0.0628878	8	5.09E-05
GJA3	5'UTR,1stExon,TSS200,TSS1500	chr13:20734889-20735819	0.0019685	0.0584933	8	0.0001999
GPC2,STAG3	1stExon,5'UTR,TSS1500,TSS200	chr7:99774935-99775862	5.23E-05	0.1000875	15	2.36E-06
GPR160	5'UTR	chr3:169781404-169782079	0.001658	-0.0903578	7	0.00019
GPR26	TSS1500,TSS200,1stExon,Body	chr10:125425262-125426598	0.0005937	0.0638187	8	1.08E-06
GPR75,LOC100302652	5'UTR,Body,1stExon,TSS200,TSS1500	chr2:54086854-54087552	8.82E-06	0.0507176	13	6.09E-06
GPR78	TSS1500,TSS200,1stExon,5'UTR	chr4:8581650-8582565	0.0007195	0.0690817	10	0.0003046
GREB1L	TSS1500,TSS200,5'UTR	chr18:18821913-18822637	0.0013917	0.0987324	5	6.38E-05
GREM1	TSS200,5'UTR	chr15:33010143-33011441	0.0004542	0.0584483	6	5.45E-05
GRIA2	TSS1500,TSS200,1stExon,5'UTR,Body	chr4:158141076-158144020	0.0012169	0.1134828	14	3.25E-05
GRIK2	TSS1500,TSS200,1stExon,5'UTR,Body	chr6:101846409-101847706	1.32E-05	0.1035775	19	5.01E-08
GRM1	5'UTR,Body	chr6:146349312-146351044	0.0007359	0.0836277	14	6.37E-08
GSC	3'UTR,Body,1stExon,TSS200	chr14:95234658-95236615	0.0003947	0.0750995	15	2.67E-08
GSX1	TSS1500,TSS200,5'UTR,1stExon,Body	chr13:28366427-28367741	0.0016672	0.062689	11	1.26E-05
GUCY1A2	Body,1stExon,5'UTR,TSS200,TSS1500	chr11:106888299-106889745	0.0015704	0.0600149	12	3.21E-05
HAMP	TSS1500,TSS200,1stExon,5'UTR,Body	chr19:35772657-35773718	0.0001962	-0.0634566	8	0.0001446
HAND2,NBLA00301	Body,1stExon,TSS1500,5'UTR,TSS200	chr4:174449827-174452985	0.0005012	0.072041	25	9.17E-14
HGFAC	TSS1500,TSS200,1stExon,Body	chr4:3443025-3444147	0.0013311	-0.0606065	8	0.0002567
HIST3H2BB,HIST3H2A	TSS1500,1stExon,3'UTR,5'UTR,TSS200	chr1:228644750-228646392	2.63E-05	0.0791908	21	2.04E-09
HLA-DMA	3'UTR,Body,1stExon,5'UTR,TSS200,TSS1500	chr6:32916606-32921791	0.0030003	-0.1006273	30	1.53E-06
HOXA10	Body,1stExon,5'UTR,TSS200,TSS1500	chr7:27213610-27214383	3.37E-06	0.0854321	11	2.59E-06
HOXA11AS,HOXA11	TSS1500,Body,1stExon,5'UTR,TSS200	chr7:27223682-27226148	0.0002176	0.0868793	37	6.56E-10
HOXA4	Body,1stExon,5'UTR,TSS200	chr7:27169674-27170554	0.0028933	0.0952171	10	0.0002406
HOXA7	Body,1stExon,5'UTR,TSS200,TSS1500	chr7:27195602-27196825	7.37E-05	0.0481773	15	4.24E-06
HOXA9	Body,1stExon,5'UTR,TSS200,TSS1500	chr7:27204349-27206544	0.0002912	0.0609225	21	9.70E-10
HOXC11	5'UTR,1stExon,Body	chr12:54367008-54368639	0.0019098	0.1107702	8	0.0002215
HOXC13	TSS1500,TSS200,1stExon,5'UTR,Body	chr12:54332026-54333823	0.0005165	0.0790446	18	7.49E-09
HOXC9	TSS1500,TSS200,5'UTR,1stExon	chr12:54393100-54394212	0.0007819	0.0791041	17	8.65E-05
HOXD1	TSS1500,TSS200,1stExon,5'UTR,Body	chr2:177052486-177054306	0.0003084	0.0680417	16	9.08E-08
HOXD11	TSS1500,TSS200,1stExon,Body	chr2:176971304-176973275	0.0004993	0.0913825	14	4.18E-08
HOXD12	TSS1500,TSS200,1stExon,Body	chr2:176963948-176965435	0.0010125	0.0775333	11	1.47E-05
HOXD13	TSS1500,TSS200,1stExon	chr2:176956841-176958174	0.0008262	0.0666303	8	1.36E-07
HOXD3	5'UTR	chr2:177029073-177030228	0.0009339	0.0731239	9	2.98E-05
HOXD8	TSS1500,TSS200,5'UTR,1stExon	chr2:176993841-176995088	0.0003154	0.096536	9	6.94E-06
HOXD9	TSS1500,TSS200,5'UTR,1stExon	chr2:176986460-176987918	0.0010119	0.0890187	12	7.71E-05
HS3ST2	TSS1500,TSS200,1stExon	chr16:22825621-22826243	7.32E-05	0.0400371	7	2.77E-05
HTR4	1stExon,5'UTR,TSS200,TSS1500	chr5:148033708-148034578	1.84E-07	0.075648	7	3.59E-09
ID2	TSS200,1stExon,Body,3'UTR	chr2:8821991-8823090	0.0003684	0.0454596	7	1.30E-06
ID4	TSS1500,TSS200,1stExon,5'UTR,Body,3'UTR	chr6:19837015-19839039	0.0002849	0.0716015	11	7.48E-05
IGF1R	TSS1500	chr15:99190447-99192228	0.0005901	0.0825031	9	1.70E-05
IGFBP3	Body,1stExon,5'UTR,TSS1500	chr7:45960243-45961126	0.0007813	0.0525848	7	3.20E-05
IGFBP7	Body,1stExon,TSS200,TSS1500	chr4:57975793-57976944	0.0009618	0.0795851	12	1.95E-06
IL18BP	TSS200,5'UTR,TSS1500,1stExon,Body	chr11:71710614-71711087	7.98E-05	-0.0727978	6	4.34E-05
IL21R	TSS1500,TSS200,1stExon,5'UTR	chr16:27413459-27414418	0.001138	-0.0848463	7	0.0001868
INS-IGF2,IGF2AS,IGF2	Body,TSS1500,5'UTR,TSS200,1stExon	chr11:2160540-2162211	0.0003075	0.0582788	34	9.87E-10
IRX1	TSS1500,TSS200,1stExon,5'UTR,Body	chr5:3595106-3597487	0.0015176	0.0587989	9	7.12E-07
IRX2,C5orf38	Body,TSS1500,1stExon,5'UTR,TSS200	chr5:2750758-2752882	0.0005213	0.096147	15	2.52E-08
IRX3	Body,TSS200	chr16:54318044-54320430	0.0007433	0.0662902	10	1.64E-07
IRX4	5'UTR,1stExon,TSS200,TSS1500	chr5:1882775-1883954	0.0012816	0.0633901	8	3.61E-05
IRX5	TSS1500,TSS200,1stExon,Body	chr16:54962385-54965492	0.0001786	0.0940876	18	1.83E-06
ISM1	TSS1500,TSS200,1stExon,Body	chr20:13200069-13202702	0.0001021	0.0897299	33	6.71E-17
JAKMIP1	5'UTR,1stExon,TSS200	chr4:6201080-6202384	0.0021627	0.0872087	7	4.28E-06
KALRN	Body,TSS1500,TSS200,1stExon,5'UTR	chr3:124303035-124303745	0.0001285	-0.0697307	6	3.34E-06
KCNA4	1stExon,5'UTR,TSS200,TSS1500	chr11:30038286-30039031	0.0015584	0.0740558	13	0.0003412
KCNB1	1stExon,5'UTR,TSS200,TSS1500	chr20:48098642-48099890	3.70E-06	0.0758459	10	3.02E-08
KCNC1	TSS1500,TSS200	chr11:17756435-17757342	2.13E-05	0.0575696	7	1.81E-06
KCNH1	Body,1stExon,5'UTR	chr1:211306365-211307408	0.0020124	0.0486658	6	0.0002249
KCNIP4	5'UTR,1stExon,TSS200,TSS1500	chr4:21949853-21951046	0.00012	0.0769488	10	1.27E-07
KCNK1	TSS1500,TSS200,1stExon	chr1:233749295-233750184	0.000523	0.1089451	9	0.0001311
KCNMA1	1stExon,5'UTR,TSS1500	chr10:79397346-79398415	6.29E-05	0.061962	6	3.31E-06
KCNN2	TSS1500,TSS200,1stExon,5'UTR	chr5:113697487-113698847	0.0010016	0.0783295	12	0.0001995
KCNQ1DN	TSS1500,TSS200,Body	chr11:2889809-2891495	4.06E-05	0.0910798	39	5.08E-14
KCNS2	TSS1500,TSS200,1stExon,5'UTR,Body	chr8:99438942-99440522	0.0013256	0.0807567	10	4.17E-05
KIAA0226,MIR922	3'UTR,Body,TSS200,TSS1500	chr3:197401134-197402207	0.0021652	-0.0675721	8	1.33E-06
KIAA1462	5'UTR,1stExon,TSS200,TSS1500	chr10:30346661-30348942	0.0003382	-0.0956962	10	4.66E-07
KIAA1755	Body,5'UTR,1stExon,TSS200,TSS1500	chr20:36888790-36889389	7.62E-05	0.0465782	10	3.15E-05
KIAA1949	Body,1stExon,5'UTR	chr6:30652647-30653799	0.0007141	-0.0469267	16	3.71E-05
KIF26B	TSS1500,TSS200,1stExon,5'UTR,Body	chr1:245317789-245319431	0.0021657	0.0602022	12	0.0001777
KIF6	Body,1stExon,5'UTR,TSS200,TSS1500	chr6:39692382-39693479	0.000278	0.091866	11	4.47E-06
KY	Body,5'UTR,1stExon,TSS200,TSS1500	chr3:134369339-134370241	0.0001359	0.0465074	13	3.73E-05
LAMA1	Body,1stExon,5'UTR,TSS1500	chr18:7116977-7118122	0.0019815	0.0504984	7	3.39E-05
LBX1,FLJ41350	3'UTR,Body,TSS1500,1stExon	chr10:102986808-102988389	0.0012785	0.0623067	9	1.56E-05
LEP	TSS1500,TSS200,1stExon,5'UTR	chr7:127880619-127881440	0.0003079	0.0756039	8	2.18E-05
LEPR	5'UTR	chr1:65991176-65991765	6.55E-05	0.1206495	5	1.96E-05
LGI1	TSS1500,TSS200,5'UTR,1stExon	chr10:95516788-95517895	0.0014525	-0.0485542	10	1.34E-05
LHFPL4	Body,5'UTR	chr3:9593701-9595269	0.0008853	0.0880461	6	8.92E-07
LMX1A	5'UTR,1stExon,TSS200,TSS1500	chr1:165324810-165325833	0.0013028	0.0441765	10	5.81E-05
LOC100128811,GPR158	Body,TSS1500,TSS200,1stExon,5'UTR	chr10:25463757-25464825	0.000231	0.0608461	15	0.0001716
LOC100190940	Body,TSS200,TSS1500	chr12:130526797-130527212	5.85E-06	0.080602	9	4.47E-06
LOC100192379,TMEM155	TSS200,5'UTR,1stExon,Body,TSS1500	chr4:122685641-122687552	0.0014351	0.0984311	14	2.03E-08
LOC100270746,C6orf41	Body,TSS200,TSS1500	chr6:26987575-26988289	0.000748	0.0822659	10	0.0003185
LOC401463,BHLHE22	Body,TSS200,TSS1500,1stExon,5'UTR	chr8:65489554-65492936	0.0008454	0.0563826	31	9.72E-06
LOC440925,SP5	Body,TSS1500,TSS200,5'UTR,1stExon,3'UTR	chr2:171570838-171574141	0.0006977	0.0760355	24	6.42E-08
LPPR4	TSS1500,TSS200,1stExon,Body	chr1:99729460-99730419	0.0003435	0.0895046	11	5.08E-05

LRIG3	TSS200,Body,1stExon,TSS1500,5'UTR	chr12:59313488-59314530	0.0008719	0.0563103	11	7.52E-06
LRP12	Body,1stExon,TSS1500	chr8:105601036-105601862	8.76E-05	0.0775543	7	1.71E-06
LTA	TSS1500,TSS200,1stExon,5'UTR,Body	chr6:31539539-31540750	2.25E-05	-0.0874996	20	1.10E-06
LVRN	TSS200,1stExon,Body	chr5:115297989-115299475	0.0013441	0.0541138	8	1.80E-06
LYPD1	5'UTR,Body	chr2:133426129-133427427	0.0017395	0.0901548	6	6.22E-05
MACROD2	TSS1500,TSS200,1stExon,5'UTR,Body	chr20:13975439-13977067	0.0010996	0.0811033	13	4.54E-06
MAFB	1stExon,5'UTR,TSS1500	chr20:39316634-39318984	0.0005202	0.0802652	10	4.46E-09
MAGI2	Body,1stExon,5'UTR,TSS200,TSS1500	chr7:79081455-79083997	0.0029949	0.0422434	23	2.40E-05
MAL	TSS1500,5'UTR,1stExon,Body	chr2:95691051-95692524	0.0012422	0.0886768	10	2.90E-06
MAL2	TSS1500,TSS200,1stExon,Body	chr8:120219902-120221268	8.78E-05	0.0753556	12	6.12E-09
MARCH11	Body,1stExon,5'UTR,TSS200,TSS1500	chr5:16179012-16181039	0.0003822	0.1096265	19	9.57E-12
MBOAT2	5'UTR,1stExon,TSS200,TSS1500	chr2:9143747-9144505	0.0014361	0.0419798	7	0.0003136
MDFIC	TSS1500,TSS200,5'UTR,1stExon,Body	chr7:114561804-114562711	0.0007859	-0.0770091	7	2.54E-08
ME1	Body,5'UTR,1stExon,TSS200,TSS1500	chr6:84140564-84141234	0.0004217	0.0485661	8	0.0003091
MEIS1	TSS1500	chr2:66660175-66661516	0.001779	0.0584456	8	2.96E-05
MEIS2	TSS200,Body,5'UTR,1stExon,TSS1500	chr15:37390666-37392106	4.20E-05	0.059458	9	1.27E-07
MIMT1,PEG3,ZIM2	TSS1500,5'UTR,1stExon,TSS200,Body	chr19:57351322-57352584	0.002184	0.0530864	17	0.0003229
MIPOL1	TSS1500,TSS200,1stExon,5'UTR	chr14:37666810-37667261	0.0006127	0.0686674	7	0.0004694
MIR124-2	TSS1500,TSS200,Body	chr8:65290848-65291990	0.0005826	0.0658982	11	0.0002901
MIR129-2	TSS1500,TSS200,Body	chr11:43601125-43603572	0.0008285	0.0807944	18	1.44E-05
MIR196B	Body,TSS200,TSS1500	chr7:27208885-27209828	9.84E-06	0.0665468	14	1.57E-06
MIR203	TSS1500,TSS200,Body	chr14:104582743-104584098	0.0004597	0.0543263	10	1.97E-06
MIR346,GRID1	Body,TSS200,TSS1500	chr10:88024529-88025555	0.0014536	-0.0871009	9	0.0004113
MKX	Body,5'UTR,1stExon,TSS200,TSS1500	chr10:28031957-28035208	0.0018274	0.1263214	19	1.06E-05
MME	TSS1500,TSS200,1stExon,5'UTR	chr3:154796701-154798079	0.0001556	0.0782099	17	2.39E-07
MSC	Body,1stExon,5'UTR,TSS200,TSS1500	chr8:72754953-72757004	0.0003029	0.0548633	17	3.64E-09
MSLNL	Body,TSS200,TSS1500	chr16:832683-833250	0.0004461	-0.0879809	10	0.0003662
MSX1	TSS1500	chr4:4858340-4860617	0.00187	0.0716469	22	0.0001158
MTNR1A	Body,1stExon,TSS200,TSS1500	chr4:187475669-187477310	0.0003334	0.0852928	15	3.30E-13
MTNR1B	TSS1500,TSS200,1stExon,Body	chr11:92702373-92703185	0.0005189	0.0767394	9	0.0003351
MYADM	TSS1500,5'UTR,TSS200,1stExon	chr19:54371775-54373043	7.16E-07	0.0825993	17	1.53E-12
MYADM	TSS1500,TSS200,5'UTR	chr19:54369015-54370016	1.17E-10	0.0928694	11	3.36E-12
MYO10	Body,1stExon,5'UTR,TSS200,TSS1500	chr5:16935695-16936927	2.37E-05	0.0514242	15	1.83E-07
MYOM2	5'UTR,1stExon	chr8:1993157-1993987	0.002646	-0.0475908	5	0.0002644
MYRIP	TSS1500,TSS200,1stExon,5'UTR	chr3:39850280-39851931	9.64E-05	0.0616136	12	2.43E-06
NALCN	5'UTR,TSS200,TSS1500	chr13:102068234-102069169	0.0016579	0.0600919	10	3.31E-05
NAT14,SSC5D	Body,3'UTR,TSS1500	chr19:55997780-55998931	2.08E-05	-0.0824149	5	1.71E-07
NAV2,LOC100126784	Body,TSS1500,TSS200,5'UTR,1stExon	chr11:19734490-19735276	0.000167	0.0469207	9	0.0001075
NCKAP1	1stExon,5'UTR,TSS200,TSS1500	chr2:183902764-183903770	4.43E-05	0.060509	12	2.01E-06
NEFL	Body,1stExon,5'UTR,TSS200,TSS1500	chr8:24812784-24814393	0.0012945	0.0758937	9	7.39E-05
NEFM	TSS1500,1stExon,TSS200,Body,5'UTR	chr8:24771645-24773268	0.0005382	0.0696214	13	2.36E-05
NELL1	TSS1500,TSS200,5'UTR,1stExon,Body	chr11:20690223-20691823	5.66E-07	0.0510131	14	3.64E-12
NELL2	Body,5'UTR,1stExon,TSS1500,TSS200	chr12:45269992-45270896	0.000137	0.0458669	19	6.38E-06
NEUROG1	1stExon,5'UTR,TSS200,TSS1500	chr5:134870977-134872649	1.78E-05	0.0622242	26	2.22E-14
NFIA	1stExon,Body,TSS1500,TSS200,5'UTR	chr1:61547632-61549542	0.0015452	0.0769214	14	3.75E-06
NGF	5'UTR,TSS200,TSS1500	chr1:115880072-115881544	0.0013357	0.0922822	10	3.95E-05
NHLH2	3'UTR,Body,5'UTR	chr1:116380572-116381660	0.0011036	0.0646277	10	3.83E-05
NKAIN3	TSS1500,1stExon,5'UTR	chr8:63160746-63161676	0.001283	0.0448562	9	3.92E-05
NKX2-2	3'UTR,Body,1stExon,5'UTR,TSS200,TSS1500	chr20:21492558-21495363	0.0014177	0.075527	14	4.50E-05
NKX2-4	1stExon,5'UTR,TSS200,TSS1500	chr20:21377671-21378560	0.0006208	0.077604	11	4.68E-05
NKX6-1	Body,1stExon,TSS200,TSS1500	chr4:85417612-85420487	0.0005626	0.0635819	19	5.86E-08
NKX6-2	3'UTR,Body,1stExon,TSS1500	chr10:134598352-134600357	0.0022192	0.0911462	17	0.0003277
NLGN1	TSS1500	chr3:173115252-173115905	0.0019822	0.0560579	7	0.0004621
NOS1	5'UTR	chr12:117798077-117799083	0.0007115	0.0511765	6	5.12E-06
NPBWR1	TSS1500,TSS200,1stExon	chr8:53852030-53854172	0.0007962	0.0502552	12	1.02E-06
NPHS2	Body,1stExon,5'UTR,TSS200,TSS1500	chr1:179544584-179545458	0.0012886	0.0657631	12	0.0002931
NPR3	TSS1500,TSS200,5'UTR,1stExon	chr5:32711095-32712623	0.0013228	0.0496917	8	1.53E-05
NPY	TSS1500,TSS200,5'UTR,1stExon	chr7:24323128-24324435	0.000726	0.0650488	11	9.36E-06
NR2F2,MIR1469	TSS1500,Body,TSS200,1stExon,5'UTR	chr15:96873184-96876737	0.0005829	0.100746	22	1.24E-07
NRG1	TSS1500,Body,1stExon,5'UTR	chr8:32405427-32406928	7.95E-05	0.1273056	8	2.81E-08
NRG1	TSS1500,TSS200,5'UTR,1stExon,Body	chr8:31496644-31498256	0.0007204	0.0906399	9	1.30E-10
NRK	TSS1500,TSS200,5'UTR,1stExon,Body	chrX:105065879-105066912	0.0027634	0.0707265	8	0.0004622
NTF3	TSS1500,TSS200,1stExon,5'UTR,Body	chr12:5540426-5541814	0.0001054	0.0550386	9	7.11E-08
NTM	TSS1500,Body,1stExon,5'UTR	chr11:131779750-131781257	0.0012022	0.0764428	15	1.32E-05
OLIG1	TSS1500,TSS200,1stExon,5'UTR	chr21:34442160-34443010	5.66E-06	0.0489017	11	2.31E-06
OLIG2	TSS1500,TSS200,1stExon,5'UTR	chr21:34397654-34398849	0.0009441	0.0459243	12	0.0001887
OLIG3	1stExon,3'UTR,5'UTR,TSS200	chr6:137814078-137815699	0.0025604	0.1274762	11	6.08E-07
OPRK1	Body,5'UTR,1stExon,TSS200,TSS1500	chr8:54162969-54164442	0.0006344	0.078302	11	2.31E-09
OR2H1	5'UTR,Body,3'UTR	chr6:29429346-29430900	0.0013668	-0.0807636	6	0.0001667
OSBPL1A	5'UTR,1stExon,TSS200,TSS1500	chr18:21977340-21978221	3.73E-06	0.0683451	10	1.99E-06
OTP	Body,5'UTR,1stExon,TSS200,TSS1500	chr5:76934327-76935719	0.0005045	0.0692008	9	2.42E-07
P2RY1	5'UTR,1stExon	chr3:152553061-152554195	0.0041306	0.0444648	7	6.28E-05
PARVA	TSS1500,TSS200,1stExon,5'UTR,Body	chr11:12398874-12399973	0.001606	0.0897316	11	5.01E-06
PAX1	TSS1500,TSS200,1stExon,5'UTR,Body	chr20:21685401-21686728	0.0004456	0.0710297	15	1.41E-09
PAX3,CCDC140	Body,TSS1500,TSS200,5'UTR,1stExon	chr2:223161771-223165320	0.0014023	0.0661243	26	1.59E-06
PAX6	5'UTR,1stExon,TSS200,TSS1500	chr11:31838674-31840698	0.0003459	0.082963	12	8.59E-08
PCDH10	TSS1500,TSS200,1stExon,5'UTR	chr4:134068592-134070819	0.0004106	0.0791529	18	4.71E-07
PCDH8	1stExon,5'UTR,TSS200,TSS1500	chr13:53422381-53423262	2.29E-05	0.0783108	10	1.29E-05
PCDH8	Body,1stExon	chr13:53419596-53420425	0.0025476	0.0600981	7	0.0001202
PCDHA7,PCDHAC1,PCDHA12,PCD	Body,TSS1500,TSS200,1stExon,5'UTR	chr5:140305713-140306458	0.0004606	0.085136	11	0.0002996
PCDHAC2,PCDHA7,PCDHA12,PCD	1stExon,Body,TSS1500,5'UTR,TSS200	chr5:140345966-140346921	0.0004146	0.0554307	11	0.0001134
PCDHGA2,PCDHGA4,PCDHGA1,P	Body,TSS1500,TSS200,1stExon	chr5:140734543-140735545	0.0011677	0.0458101	8	0.0002494
PCDHGA4,PCDHGA11,PCDHGA9,	Body,TSS200,5'UTR,1stExon	chr5:140810051-140811312	0.0008775	0.0901336	15	7.99E-06
PDGFRA	5'UTR	chr4:55097420-55098483	0.0022177	0.0543431	7	3.04E-05
PDZRN3	1stExon,5'UTR,TSS200,TSS1500	chr3:73673893-73674702	1.01E-05	0.0671952	15	1.58E-06
PDZRN4	TSS1500,TSS200,1stExon,Body	chr12:41581414-41583115	0.0023088	0.0661572	9	2.76E-05
PHF21B	Body,1stExon,TSS200,5'UTR,TSS1500	chr22:45403493-45406130	0.0016849	0.0712387	22	4.60E-09

PHOX2A	Body,1stExon,5'UTR,TSS200,TSS1500	chr11:71954381-71955599	0.0004423	0.0678805	12	4.36E-05
PHYHIPL	TSS1500,TSS200,5'UTR,1stExon,Body	chr10:60935663-60937501	0.0005389	0.1158522	18	1.13E-08
PITX2	Body,1stExon,5'UTR,TSS200,TSS1500	chr4:111542825-111545119	0.0004492	0.0785675	14	1.91E-06
PIWIL1	5'UTR	chr12:130823570-130824403	0.002658	0.0668072	6	0.0001568
PKDREJ	1stExon,TSS200,TSS1500	chr22:46658960-46659595	0.00165	0.0644954	5	0.0001056
PLA2G7	5'UTR,1stExon,TSS200,TSS1500	chr6:46702683-46704077	1.06E-08	0.0745723	19	5.23E-14
PLAGL1	TSS1500	chr6:144386231-144387124	0.0016097	-0.0566848	12	0.0002576
PLCXD3	Body,1stExon,5'UTR,TSS200,TSS1500	chr5:41509849-41511150	0.0018731	0.064435	13	0.0004938
PLEKHH2	TSS1500,TSS200,1stExon,5'UTR	chr2:43863985-43865055	0.0002601	0.0697465	12	1.09E-07
PLOD2	Body,5'UTR,1stExon,TSS200,TSS1500	chr3:145878431-145879710	0.0010522	0.1019941	12	4.71E-06
PMP22	5'UTR,1stExon,TSS200,TSS1500	chr17:15165803-15166505	0.0009062	0.0438404	10	0.0002907
PNOC	TSS1500,TSS200,5'UTR,1stExon	chr8:28173732-28175463	0.0007258	-0.0715074	12	1.27E-07
POM121L2	1stExon,5'UTR,TSS200	chr6:27278678-27280195	0.0041562	-0.0731354	9	0.0001289
PON3	Body,1stExon,TSS200,TSS1500	chr7:95025194-95026937	7.22E-05	0.0753857	23	2.65E-12
POU4F1	Body,5'UTR,1stExon,TSS200,TSS1500	chr13:79175061-79177925	0.0038221	0.0558405	16	0.0004181
POU4F2	TSS1500,1stExon,5'UTR,Body	chr4:147557774-147561775	0.0005717	0.1001696	16	6.64E-08
PPM1M	TSS200,1stExon,5'UTR	chr3:52280037-52280702	0.0018698	-0.0617161	7	1.41E-05
PPP2R2B	Body,5'UTR,1stExon,TSS200,TSS1500	chr5:146257484-146258785	0.0001369	0.0735972	17	2.33E-08
PRDM14	5'UTR,1stExon,TSS200,TSS1500	chr8:70982867-70984559	0.0017841	0.0783514	14	2.76E-06
PRDM5	1stExon,5'UTR,TSS200,TSS1500	chr4:121843717-121844401	5.51E-05	0.0700539	9	2.24E-05
PREX2	TSS1500,TSS200,Body	chr8:68864012-68865187	0.0001304	0.0512496	13	1.14E-05
PRKCZ	5'UTR,Body	chr1:2064691-2066981	0.0023117	-0.0796991	11	0.0003515
PRKG1	TSS1500,TSS200,1stExon,5'UTR	chr10:52750296-52751341	0.0014311	0.0572797	8	0.0002198
PRRG1	TSS1500,TSS200,1stExon,5'UTR	chrX:37208111-37209004	0.0009882	0.0829929	8	0.0001454
PRSS12	Body,1stExon,5'UTR,TSS200,TSS1500	chr4:119273361-119274837	0.0005913	0.0672207	8	5.09E-07
PRTG	Body,1stExon,5'UTR,TSS200,TSS1500	chr15:56034801-56035616	0.0005845	0.057681	9	3.83E-05
PTF1A	TSS1500,TSS200,1stExon	chr10:23480730-23481786	0.000153	0.045048	9	2.33E-05
PTGS2	Body,5'UTR,1stExon,TSS200,TSS1500	chr1:186649153-186649985	0.0002099	0.068188	13	9.37E-05
PTH2R	TSS1500,TSS200,1stExon,5'UTR,Body	chr2:209271164-209271986	0.0015136	0.0642654	8	3.49E-05
PTPRM	1stExon,5'UTR,Body	chr18:7567426-7568539	0.000329	0.0472648	8	3.29E-06
PTPRN2	TSS200,TSS1500	chr7:158380577-158381220	0.0003513	0.0598992	6	3.61E-05
PXDN	Body,1stExon,TSS200,TSS1500	chr2:1747364-1749076	0.0003072	0.0673671	10	6.24E-08
RAB32	TSS1500,TSS200,1stExon,5'UTR,Body	chr6:146864186-146865263	0.0011131	0.0619227	19	0.0001254
RAD21L1	TSS1500,TSS200,1stExon,5'UTR	chr20:1206255-1207118	0.0006863	0.071694	10	0.0002735
RADIL	5'UTR,TSS200,TSS1500	chr7:4922565-4923615	0.001661	0.0564429	7	7.77E-06
RBM24	TSS1500,TSS200,1stExon,Body,5'UTR	chr6:17280551-17282533	0.000991	0.0604994	15	8.02E-11
RELN	Body,1stExon,TSS200,TSS1500	chr7:103629370-103630549	0.0002283	0.0795104	9	2.77E-06
REM2	TSS1500,TSS200,1stExon,5'UTR	chr14:23351487-23352488	0.0010043	-0.0670851	6	1.32E-05
RNF128	TSS1500,Body,TSS200,1stExon,5'UTR	chrX:105969622-105970705	0.0006628	0.0642318	12	5.69E-05
RNF144A	TSS1500,TSS200,5'UTR,1stExon	chr2:7057153-7057945	0.0007893	0.0716665	7	0.0001612
RNF180	TSS1500,TSS200,5'UTR	chr5:63461216-63462187	5.81E-07	0.0822897	11	2.43E-08
RNF220	5'UTR	chr1:44872657-44873692	0.0010557	0.0546198	6	1.05E-06
RNU5E,RNU5D,ACOT12	Body,TSS1500	chr5:80689492-80690458	0.0011644	0.0504486	6	0.0004347
RPL13AP5,SNORD33,SNORD32A,	Body,TSS1500,TSS200	chr19:49992527-49994239	0.0012232	-0.1024304	11	1.90E-05
RPRM	3'UTR,1stExon,5'UTR,TSS200,TSS1500	chr2:154334294-154335766	0.0003595	0.070972	16	4.10E-07
RPS12,SNORD100,SNORA33	Body,TSS1500,TSS200	chr6:133137262-133138165	0.0010078	-0.0819243	5	0.000481
SALL1	Body,5'UTR,1stExon,TSS200,TSS1500	chr16:51183363-51190201	0.00046	0.1072546	47	9.14E-17
SALL3	TSS1500,TSS200,1stExon,Body	chr18:76737545-76740962	0.0006044	0.0856239	16	1.37E-07
SAMD12	Body,5'UTR,1stExon,TSS200,TSS1500	chr8:119633844-119634634	0.0012452	0.0472665	9	0.0004698
SATB2	5'UTR	chr2:200322034-200324281	0.0011868	0.0757068	10	2.41E-06
SCGB1C1,LOC653486	TSS200	chr11:192897-193062	0.0003602	-0.0476702	5	0.0003593
SCN3B	1stExon,5'UTR,TSS200,TSS1500	chr11:123524938-123525714	0.0009591	0.06204	8	1.29E-06
SEZ6	Body,1stExon,5'UTR,TSS200,TSS1500	chr17:27331976-27333617	0.0017302	0.1249496	16	5.33E-08
SFRP1	Body,1stExon,5'UTR,TSS200,TSS1500	chr8:41165144-41167278	0.0003049	0.071134	11	9.80E-06
SGEF	TSS200,1stExon,5'UTR,Body	chr3:153839142-153840536	0.0035056	0.054289	11	6.52E-05
SH3GL3	TSS1500,TSS200,Body,1stExon,5'UTR	chr15:84115529-84116935	0.0012001	0.0706488	12	8.07E-06
SH3RF3,LOC100287216	TSS1500,Body,TSS200,1stExon	chr2:109745516-109747264	0.0015	-0.0478954	15	2.04E-05
SIM2	TSS1500,TSS200,1stExon	chr21:38070148-38072186	0.0007642	0.0590438	19	7.53E-06
SIX6	TSS200,1stExon,5'UTR,Body	chr14:60975811-60977353	4.18E-05	0.0458451	11	9.83E-06
SLC18A2	TSS1500,TSS200,5'UTR,Body	chr10:119000083-119001590	0.0021728	0.0712907	12	5.06E-06
SLC25A21,LOC100129794	Body,5'UTR,1stExon,TSS200	chr14:37641436-37641964	0.0014143	0.0448904	6	8.27E-05
SLC32A1	TSS1500,TSS200,5'UTR,1stExon	chr20:37352612-37353700	0.0004453	0.054057	10	0.0001771
SLC6A1	TSS1500,TSS200,1stExon,5'UTR	chr3:11033866-11035079	0.0011555	0.0614539	11	2.35E-05
SLC6A15	5'UTR,1stExon,TSS200,TSS1500	chr12:85305742-85307424	0.0006838	0.1065045	17	3.38E-08
SLCO3A1	TSS1500,TSS200,1stExon,Body	chr15:92396240-92397873	0.0001044	0.1134648	11	1.31E-08
SLIT2	TSS1500,TSS200,Body	chr4:20254519-20256841	0.0036222	0.0790866	9	0.0001863
SLIT3	1stExon,5'UTR,TSS200,TSS1500	chr5:168727686-168729015	8.92E-05	0.1004178	13	2.67E-08
SLITRK3	5'UTR,1stExon,TSS200,TSS1500	chr3:164913828-164915196	0.0001407	0.092779	15	4.97E-08
SLITRK4	5'UTR,1stExon,TSS200,TSS1500	chrX:142722095-142723826	0.0007012	0.0813393	11	3.10E-06
SLITRK5	TSS1500,TSS200,1stExon,5'UTR	chr13:88323607-88325384	0.000812	0.0987416	12	6.28E-09
SMOC2	TSS1500,TSS200,5'UTR,1stExon,Body	chr6:168841257-168842491	0.0005375	0.0684397	11	2.94E-05
SNAP91	Body,5'UTR,TSS200,TSS1500	chr6:84418433-84419360	0.0001721	0.0619753	13	1.44E-05
SNCA	5'UTR,1stExon,TSS200,TSS1500	chr4:90757452-90759203	0.0003409	0.0775092	16	1.06E-14
SNHG3-RCC1,RCC1	Body,TSS1500,5'UTR	chr1:28843736-28844479	0.0013718	-0.0837786	5	2.82E-06
SNRPN	TSS1500,TSS200,1stExon,5'UTR	chr15:25068564-25069376	0.0003966	0.0499655	9	0.0003116
SNX7	TSS1500,TSS200,1stExon,5'UTR,Body	chr1:99126623-99127707	0.0007623	0.0556242	8	7.61E-05
SORCS3	TSS1500,TSS200,1stExon,5'UTR	chr10:106399513-106401517	9.62E-06	0.0656846	15	1.86E-10
SOX1	TSS1500,TSS200,1stExon,5'UTR,3'UTR	chr13:112720427-112723581	0.0006854	0.0982677	19	9.12E-06
SOX21	1stExon,5'UTR,TSS200,TSS1500	chr13:95363755-95365673	0.0004577	0.0698854	16	3.22E-08
SP9	TSS1500,TSS200,Body	chr2:175199259-175200477	0.001589	0.0678683	13	0.0001924
SPACA1	TSS1500,TSS200,Body	chr6:88757302-88757878	2.49E-07	0.0723759	6	1.25E-07
SPAG6	TSS1500,TSS200,1stExon,5'UTR,Body	chr10:22633916-22635028	3.82E-07	0.0957979	14	3.35E-09
SPG20	5'UTR,1stExon,TSS200,TSS1500	chr13:36919819-36921174	0.0004963	0.0667402	17	7.74E-09
SPOCK1	Body,5'UTR,1stExon,TSS200	chr5:136833893-136835107	2.87E-05	0.0731682	10	5.38E-06
STK32A	TSS1500,TSS200,5'UTR,1stExon	chr5:146613976-146614719	0.0001923	0.0747002	12	0.0001141
SULT4A1	Body,1stExon,TSS200,TSS1500	chr22:44257997-44258712	2.31E-05	0.0783042	8	7.55E-06
SUSD5	Body,5'UTR,1stExon,TSS200,TSS1500	chr3:33259920-33261112	0.0030926	0.0456819	8	0.0003818

SVIL	5'UTR,TSS200,TSS1500	chr10:30024328-30026143	0.0010882	0.053483	13	9.19E-05
SYNM	TSS1500,TSS200,1stExon,Body	chr15:99644973-99646443	0.0004743	0.0845139	11	1.33E-11
TAC1	TSS1500,TSS200,5'UTR,1stExon	chr7:97360615-97361408	0.0009156	0.0783133	9	0.0003138
TACR1	1stExon,5'UTR,TSS200,TSS1500	chr2:75425832-75428132	0.0007941	0.0497123	21	3.59E-09
TACSTD2	1stExon,5'UTR,TSS200,TSS1500	chr1:59042065-59043576	0.0011682	0.0642042	13	9.93E-07
TAP1,PSMB9	Body,TSS1500	chr6:32819921-32820691	0.0019929	-0.0813851	18	0.0002055
TBX15	5'UTR,1stExon,TSS200,TSS1500	chr1:119531625-119532773	0.0014899	0.0591491	15	0.0003084
TBX18	1stExon,TSS200,TSS1500	chr6:85473773-85474595	0.0003709	0.0968622	11	0.0001101
TBX20	1stExon,5'UTR,TSS200,TSS1500	chr7:35293245-35294658	0.0025549	0.0541147	12	0.0001062
TBX3	1stExon,5'UTR,TSS200,TSS1500	chr12:115120789-115122304	0.0010598	0.0464933	14	0.0001246
TCF15	1stExon,TSS200,TSS1500	chr20:590799-591400	0.0005289	0.0638271	7	0.0002198
TCF21	TSS1500,TSS200,1stExon,5'UTR,Body	chr6:134209935-134211217	0.0012925	0.0713098	14	0.0002027
TFAP2A	Body,1stExon,5'UTR,TSS200,TSS1500	chr6:10415077-10416373	0.0005091	0.0879842	17	2.26E-06
TIAM1	5'UTR,TSS200,TSS1500	chr21:32930938-32932073	0.0022047	0.0464479	8	0.0001029
TIGIT	TSS1500,TSS200,5'UTR,1stExon	chr3:114012316-114012912	0.0027975	-0.0862679	5	0.0002421
TJP1	Body,5'UTR,1stExon,TSS200,TSS1500	chr15:30113651-30115299	0.0004556	0.1093873	13	1.37E-06
TLL1	1stExon,5'UTR,Body	chr4:166794786-166796183	0.0002649	0.0440199	13	9.06E-06
TLX1,TLX1NB	TSS1500,5'UTR,TSS200,1stExon,Body	chr10:102890514-102891879	0.0006537	0.0685051	12	0.0001448
TLX3	TSS1500,TSS200,5'UTR,1stExon,Body	chr5:170734856-170737396	0.0009802	0.077172	17	1.35E-08
TMC8,TMC6	Body,TSS1500	chr17:76129099-76130305	0.0014848	-0.0730471	7	2.97E-05
TMEM108	TSS1500,TSS200,5'UTR,1stExon	chr3:132756721-132757973	0.0017364	0.074818	16	9.44E-05
TMEM132C	TSS1500,Body	chr12:128751042-128753103	0.0023454	0.0721881	9	2.55E-05
TMEM132E,C17orf102	TSS1500,TSS200,1stExon,5'UTR,Body	chr17:32906991-32908285	0.0023309	0.0472606	11	0.0004387
TMEM171	TSS1500,TSS200,5'UTR,1stExon	chr5:72415687-72416677	0.0005699	0.040957	11	1.27E-06
TMEM51,C1orf126	TSS1500,5'UTR,1stExon	chr1:15479792-15481367	0.0005719	0.0586905	10	4.09E-08
TRIL	1stExon,5'UTR,TSS200,TSS1500	chr7:28995458-28998595	0.0002092	0.0719436	23	4.78E-07
TRIM26	5'UTR	chr6:30174651-30176107	0.000506	-0.1092676	13	1.55E-10
TRIM58	TSS1500,TSS200,1stExon,Body	chr1:248019757-248021163	0.0001793	0.0784849	13	4.55E-12
TRPC6	Body,5'UTR,1stExon,TSS200,TSS1500	chr11:101453871-101454996	0.0021123	0.0504769	10	0.0004113
TSHZ3	TSS1500	chr19:31840737-31844182	0.0010437	0.0994063	16	1.10E-10
TSPYL5	1stExon,5'UTR,TSS200,TSS1500	chr8:98289880-98290601	0.0003626	0.0560741	10	0.0001561
TWIST1	TSS200,TSS1500	chr7:19157339-19158954	0.0004193	0.0573912	25	1.69E-12
UACA	Body,1stExon,5'UTR,TSS1500	chr15:71054899-71056145	0.0033844	0.0635194	9	0.0002376
ULBP1	TSS200,5'UTR,1stExon,Body	chr6:150285031-150286212	0.0004579	0.0528784	8	1.58E-07
UNC5D	TSS1500,TSS200,Body	chr8:35092687-35093901	0.0016298	0.1155574	6	2.88E-06
UNC80	TSS1500,TSS200,1stExon,5'UTR,Body	chr2:210636350-210637094	2.27E-06	0.1048319	9	1.00E-06
UNCX	TSS1500,TSS200,1stExon,Body	chr7:1269693-1274752	0.0010318	0.0875628	28	9.46E-09
USH1C	Body,1stExon,5'UTR,TSS200,TSS1500	chr11:17565744-17566772	0.0003662	0.0823325	12	2.50E-07
VARS	Body,5'UTR	chr6:31762353-31763174	0.0002666	-0.0493251	16	8.76E-09
VARS2,GTF2H4	TSS1500,Body,3'UTR,TSS200,1stExon,5'UTR	chr6:30881112-30882358	7.86E-05	-0.0682011	34	1.37E-13
VAX1	Body,1stExon,TSS200,TSS1500	chr10:118896516-118898082	0.0003631	0.0629859	12	7.31E-07
VIPR2	Body,1stExon,5'UTR,TSS1500	chr7:158936508-158938780	0.001883	0.0994686	12	4.73E-13
VPS52,RPS18	TSS1500,Body	chr6:33240468-33241226	0.0025746	-0.0481156	6	0.000346
VWC2	TSS1500,TSS200,1stExon,5'UTR	chr7:49812836-49813763	0.0006012	0.0702223	9	1.67E-05
WASF3	TSS1500,TSS200,5'UTR	chr13:27131484-27132431	0.0006162	0.0407078	12	0.0001044
WBSCR17	TSS1500,TSS200,1stExon,Body	chr7:70596377-70598282	0.0003946	0.07791	12	1.56E-06
WISP3	TSS1500,TSS200,1stExon,5'UTR,Body	chr6:112374427-112375870	9.57E-05	-0.0970466	8	6.67E-09
WSCD1	TSS1500,5'UTR	chr17:5973421-5974609	0.0009763	0.0764081	9	1.02E-05
WT1,WIT1	Body,TSS1500,1stExon,5'UTR,TSS200	chr11:32456069-32457878	6.75E-05	0.0840436	12	9.42E-07
ZAP70	TSS1500,TSS200	chr2:98329337-98330020	0.0002697	-0.0409983	7	0.0001661
ZFP42	TSS1500,TSS200,1stExon,5'UTR	chr4:188916496-188917251	0.000102	0.0647122	13	4.23E-05
ZIC4	Body,TSS200,TSS1500	chr3:147108512-147112096	0.0017463	0.0818397	22	1.05E-05
ZIC4,ZIC1	TSS1500,TSS200,1stExon,5'UTR	chr3:147125712-147128157	0.001063	0.091256	31	2.11E-08
ZIC5	Body,1stExon	chr13:100621382-100622790	0.0021989	0.0794807	5	0.0003844
ZNF503,C10orf41	1stExon,TSS1500,TSS200,5'UTR,Body	chr10:77160907-77162143	1.67E-05	0.0559651	13	1.66E-07
ZNF536	5'UTR	chr19:30865486-30866365	0.0004171	0.0693866	6	0.0001161

Supplementary Table 3: The 490 DMRs identified as differentially methylated during the course of treatment. Information is provided regarding the nearest gene (‘gene_assoc’), genomic feature (‘group’), genomic region (‘hg19coord’), p value (‘meanpval’), maximal methylation change at a single CpG site within the DMR (‘maxbetafc’), the number of probes incorporated within the DMR (‘no.probes’), and lowest p value at a single CpG site within the DMR (‘minpval’).

Gene	Before treatment	Relapse	P_{FDR}
MAFB	6.50	8.90	**0.0006**
FZD8	7.08	6.12	**0.0006**
DST (BEND6 DMR)	8.11	6.73	**0.0008**
SLCO3A1	6.00	6.88	**0.0008**
KCNMA1	5.15	4.36	**0.0025**
ADAMTS17	4.91	4.33	**0.0031**
DAGLA	6.88	6.41	**0.0031**
MBOAT2	7.42	6.17	**0.0031**
MYRIP	5.61	5.17	**0.0048**
HOXA4	6.39	6.01	**0.0092**
ZNF503	5.08	5.72	**0.0111**
CDK6	5.99	5.42	**0.0113**
FGF12	4.45	4.09	**0.0137**
GJA3	6.53	7.09	**0.0151**
TIAM1	6.02	5.44	**0.0181**
JAKMIP1	5.40	5.84	**0.0181**
RNF144A	6.14	5.89	0.0982
POMGNT2 (c3orf39 DMR)	5.69	5.54	0.2290
HIST3H2A	7.99	7.84	0.2515
ADRA1B	4.69	4.81	0.2716
CCSER1 (FAM190A DMR)	5.37	5.52	0.3982
FLJ42289	*Not assessed*	*Not assessed*	*Not assessed*

Supplementary Table 4: Differential expression of DMR-associated genes following patient treatment. Mean log2-transformed expression values before treatment and at relapse in paired samples from 13 patients (GSE37168). Expression could not be assessed for *FLJ42289* due to a lack of data.

Variable	p	rho
Age	0.033	0.168
Gender	0.624	-
WCC	0.079	0.213
CD38 (%)	0.967	-0.005
CD38 (low/high)	1.000	-
IGHV (M/U)	0.004	-
IGHV (% seq)	<0.001	0.344
del(11q)	0.001	-
del(13q)	0.633	-
del(17p)	0.790	-
del(17p) / *TP53* mut.	1.000	-
Trisomy 12	0.304	-
ATM mut.	0.246	-

Supplementary Table 5: Associations between *HOXA4* methylation and disease pathology in the Newcastle cohort. Associations with age, white cell count (WCC, 109/L), *CD38* expression (%) and *IGHV* sequence homology (%) were calculated by Spearman's rank correlation coefficient. Associations with gender, CD38 (low = <30%; high = >30%), *IGHV* mutation status (mutated / unmutated), cytogenetic abnormalities and ATM mutations were calculated by Fisher's exact test.

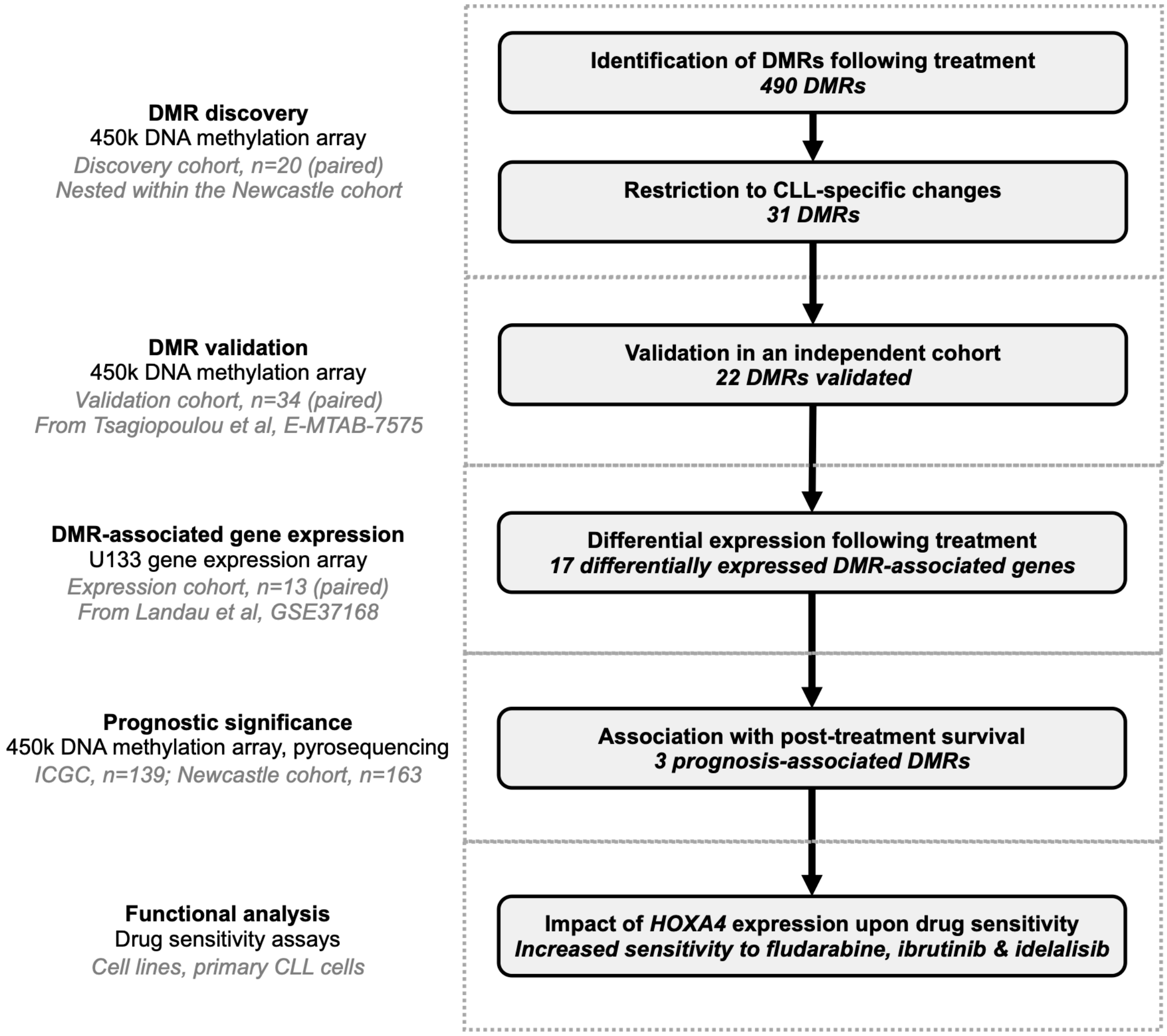

Supplementary Figure 1: Overview of the study approach. During the discovery phase, differentially methylated regions (DMRs) were identified through epigenome-wide analysis of DNA methylation in paired samples from 20 patients within the discovery cohort taken at two time-points during treatment. DMRs displaying differential methylation during the course of B-cell development were excluded, revealing 31 CLL-specific and treatment-specific DMRs. Validation was performed in an independent of 34 patients, with paired samples from prior to first treatment and at relapse. The expression of DMR-associated genes was assessed in paired samples taken from 13 patients prior to treatment and at relapse. The prognostic significance of CLL-specific differentially methylated, differentially expressed genes confirmed to display a significant correlation between methylation and expression was assessed by examining

associations with post-treatment survival in the ICGC and Newcastle cohorts. Leading candidates were taken forward for functional analysis in cell lines and primary CLL cells.

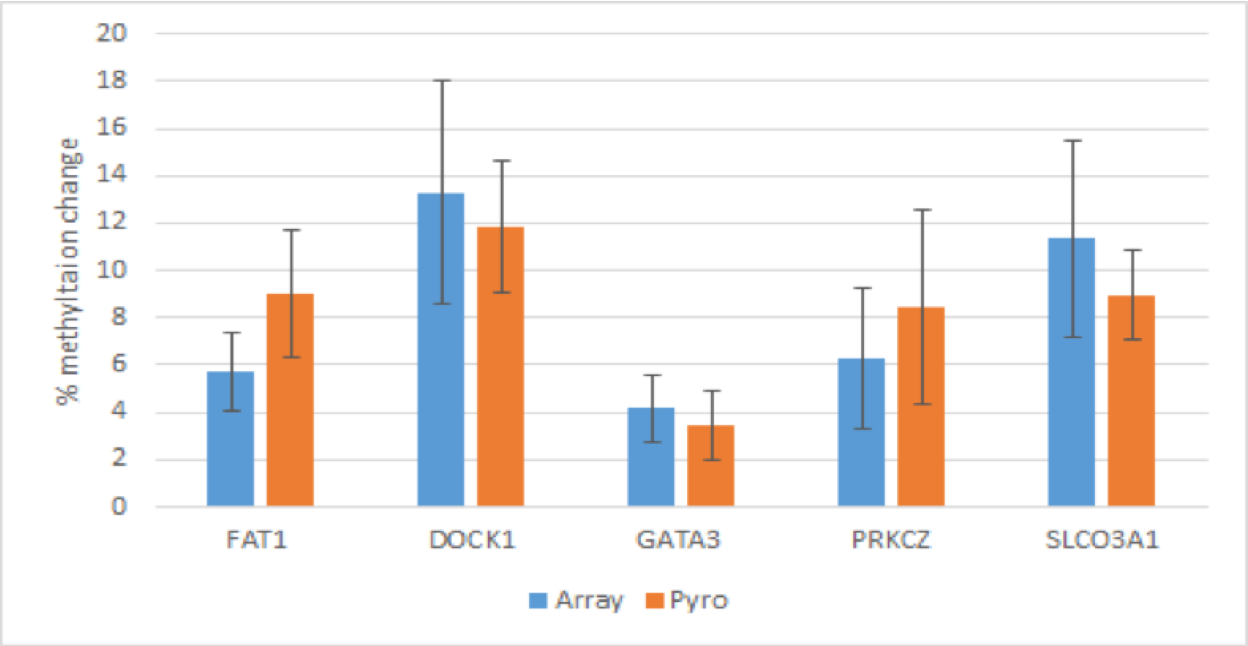

Supplementary Figure 2: Confirmation of DMRs by pyrosequencing. Average methylation changes at five representative DMRs measured by microarray (blue) and pyrosequencing (orange) in paired samples from 20 CLL patients are displayed with standard deviation.

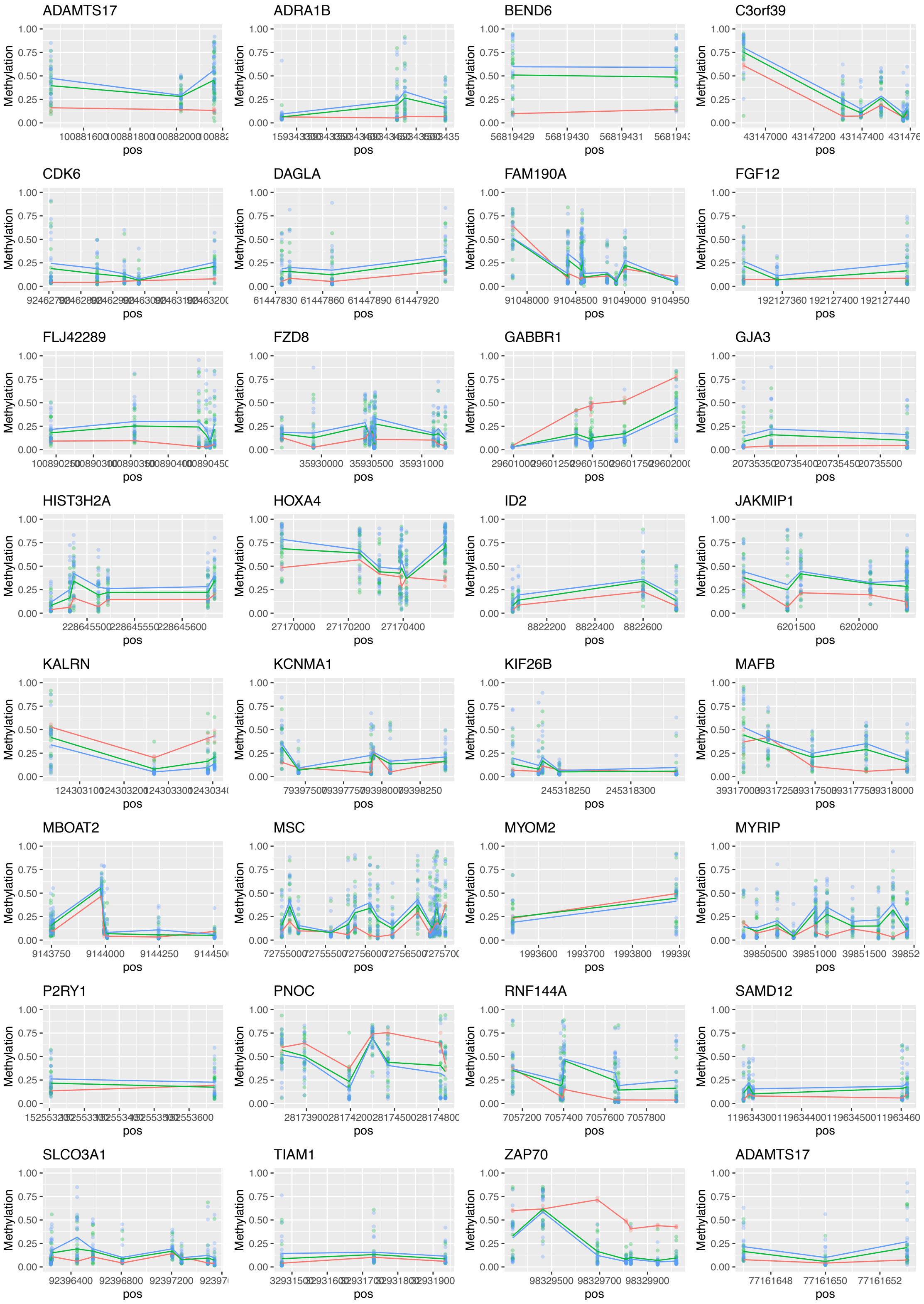

Supplementary Figure 3: DNA methylation at the 32 CLL-specific DMRs. DNA methylation (β) by genomic position across each of the DMRs, measured at the A (green) and B (blue) timepoints in the 20 patients from the discovery cohort, and in memory B-cells (red). Lines indicate mean values, with individual patient values plotted.

ADAMTS17

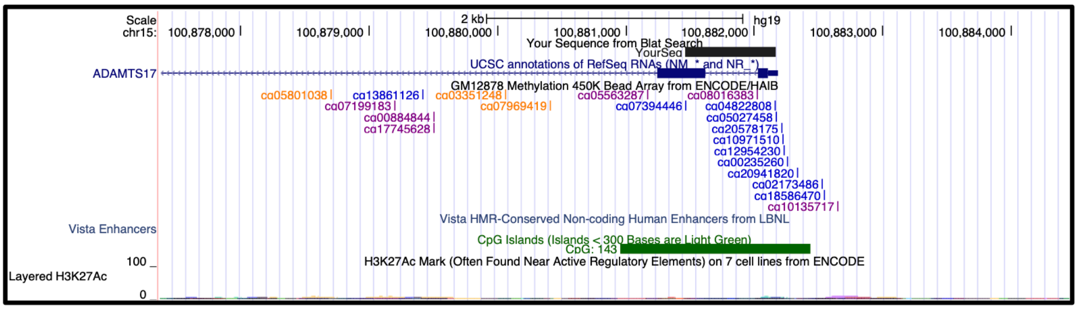

BEND6

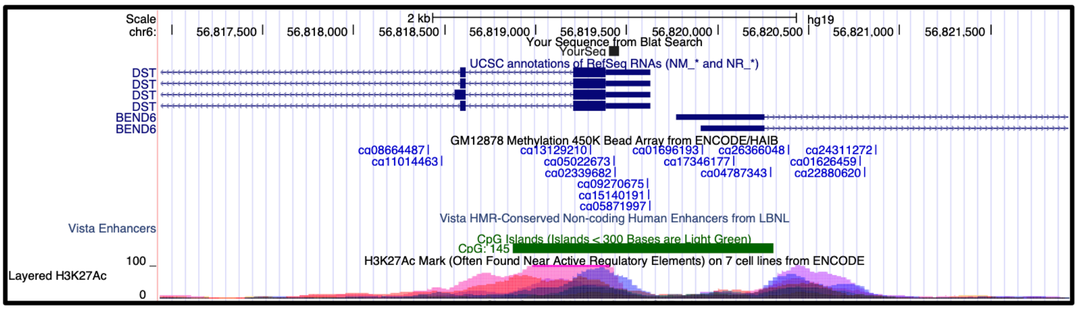

CDK6

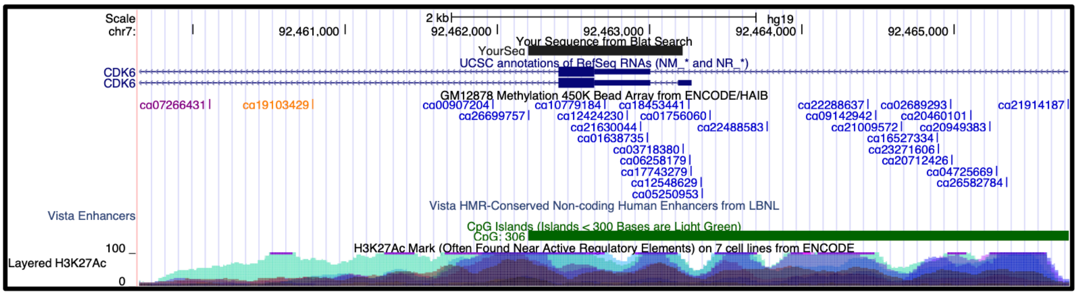

DAGLA

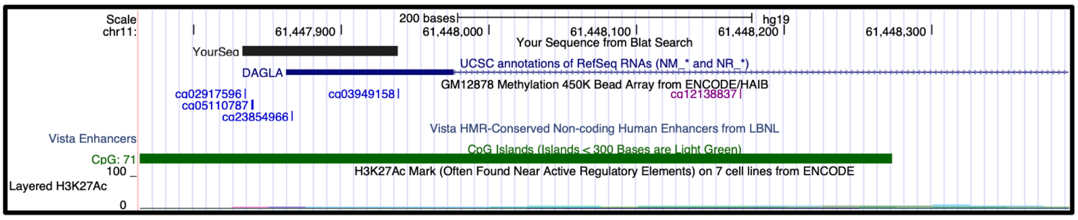

FGF12

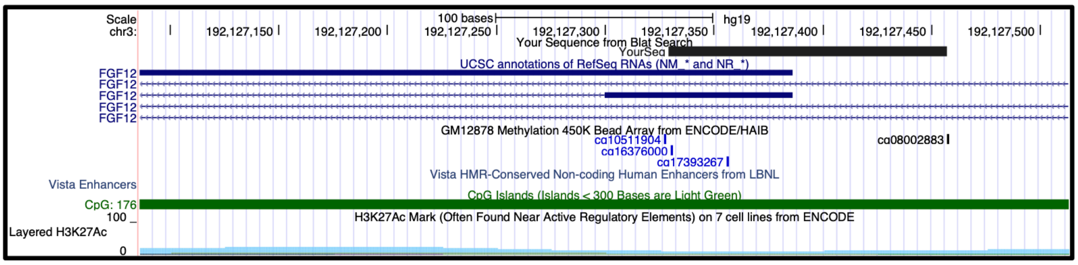

FZD8

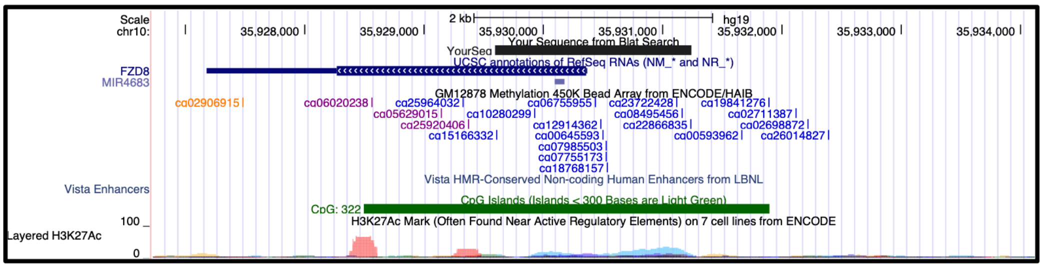

GJA3

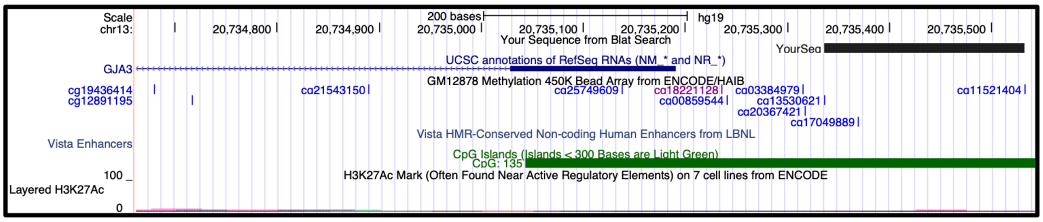

HOXA4

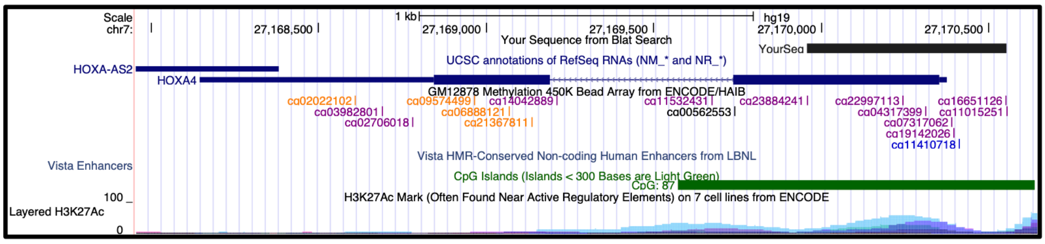

JAKMIP1

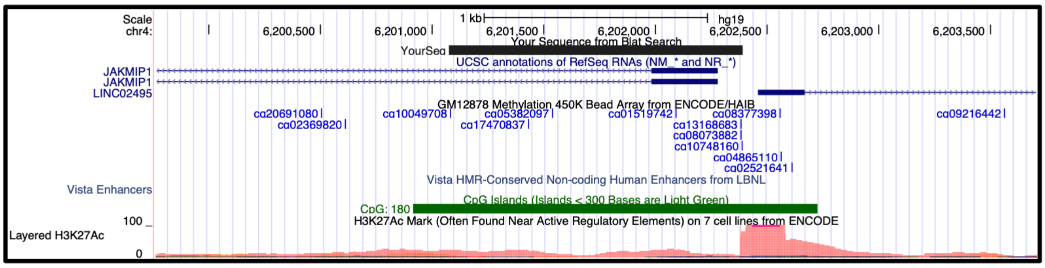

KCNMA1

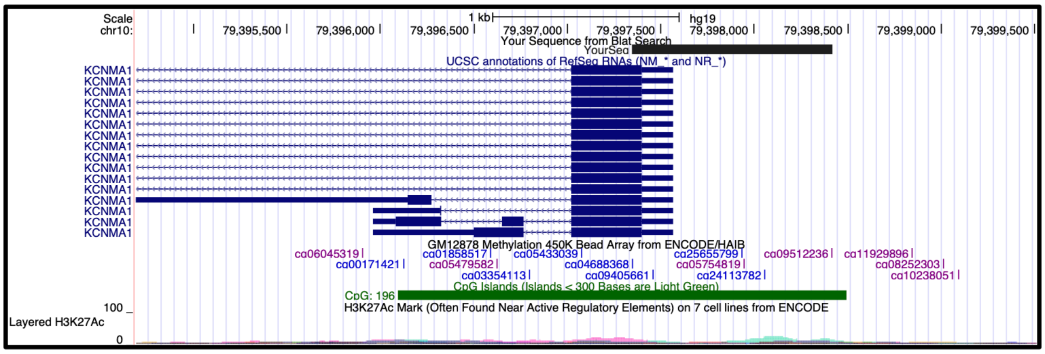

MAFB

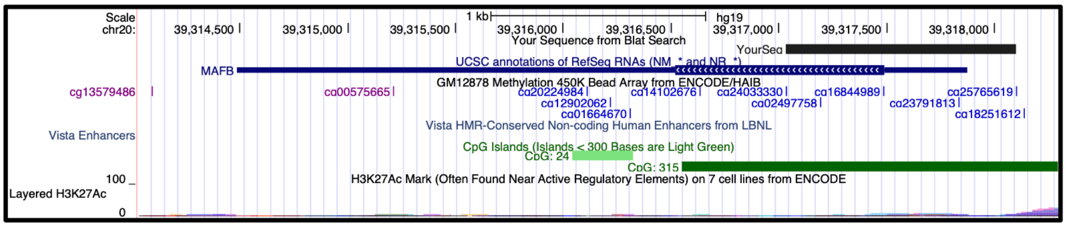

MBOAT2

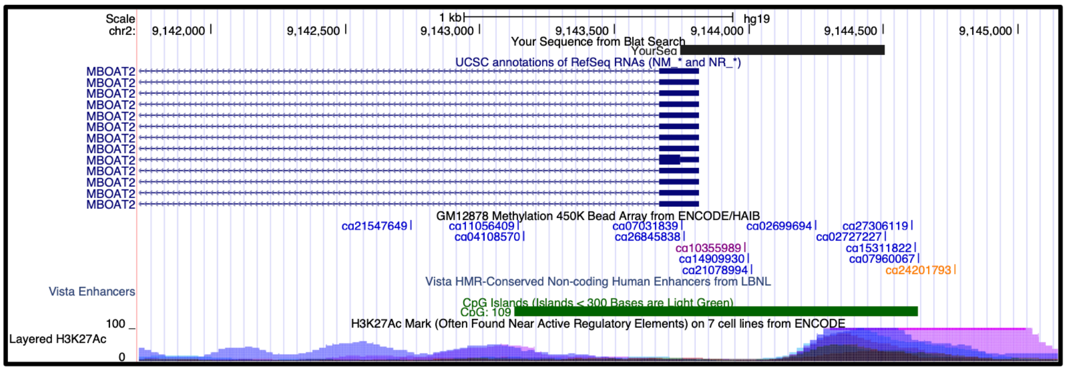

MYRIP

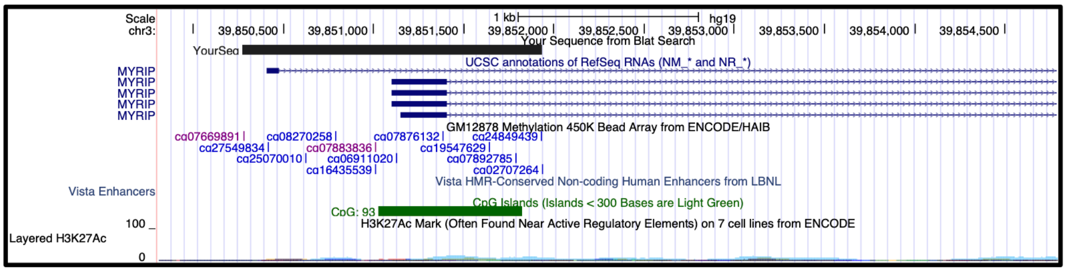

RNF144A

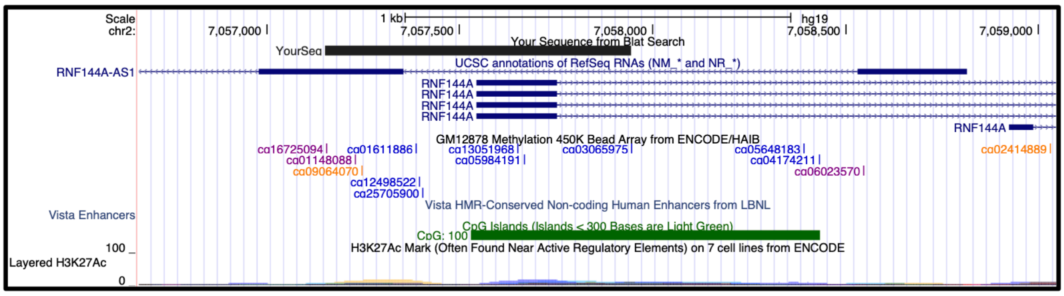

SLCO3A1

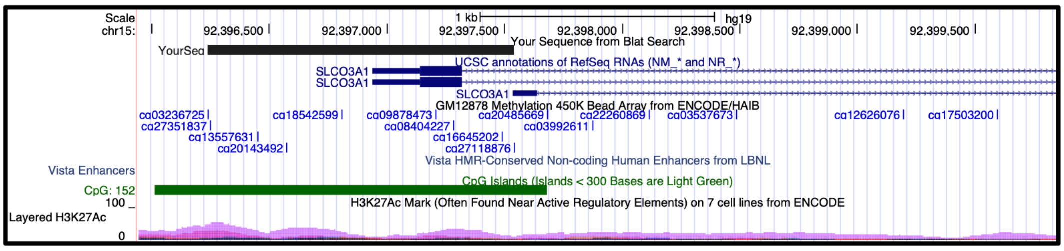

TIAM1

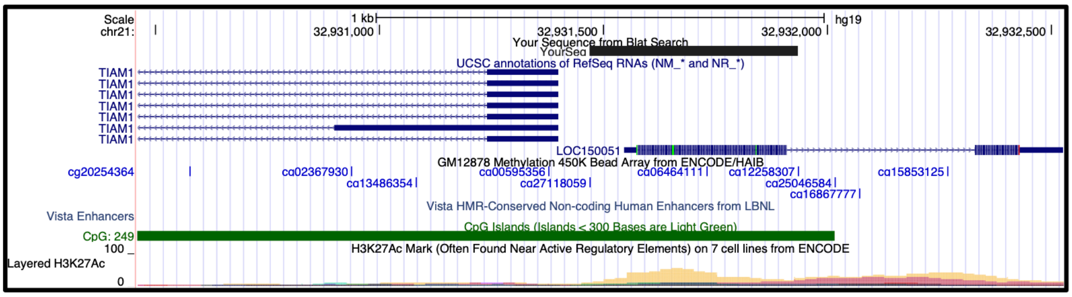

ZNF503

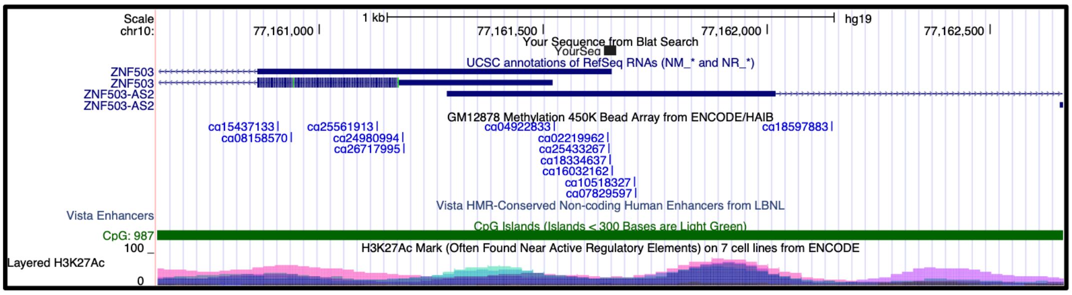

Supplementary Figure 4: Mapping of DMRs displaying differential expression following treatment. Images taken from UCSC Genome Browser for the 17 DMRs identified as showing differential expression following therapy. DMRs are indicated as black rectangles. Further annotation is provided for gene structure, the mapping of Illumina Human Methylation450 microarray probes, CpG islands, and H3K27 acetylation data from the ENCODE Project.

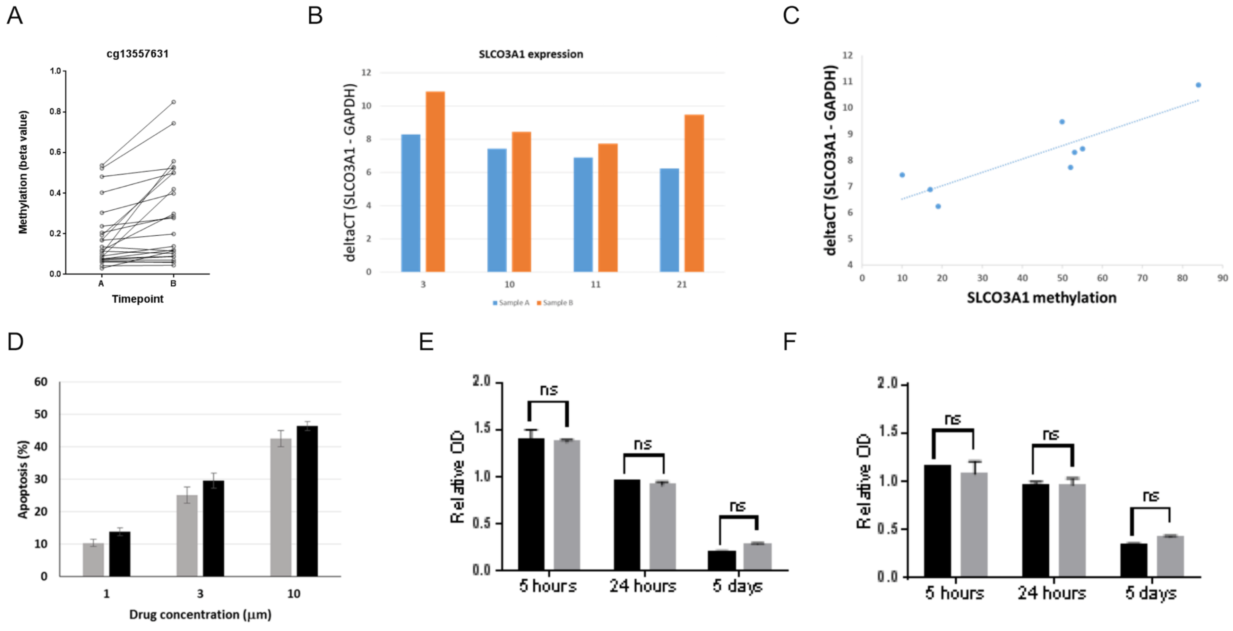

Supplementary Figure 5: Re-expression of *SLCO3A1* does not sensitise leukaemic cell lines to chemotherapy. A: methylation (β) at cg13557631 in 20 patients by time-point. B: *SLCO3A1* expression in paired samples from four patients (A, blue; B, orange), measured by RT-qPCR. C: correlation of DNA methylation and gene expression in eight paired-samples from four patients, measured by pyrosequencing and RT-qPCR respectively. D: apoptosis in control (grey) and *SLCO3A1*-expressing (black) NALM6 cells in response to fludarabine exposure, measured at 48 hours after drug treatment by detection of Annexin V by flow cytometry. E-F: apoptosis in control (black) and *SLCO3A1*-expressing (grey) SEM cells in response to 1 μM (E) and 0.5 μM (F) fludarabine exposure for 5 hours, 24 hours or 120 hours, measured at 48 or 120 hours after drug treatment by detection of Annexin V by flow cytometry.

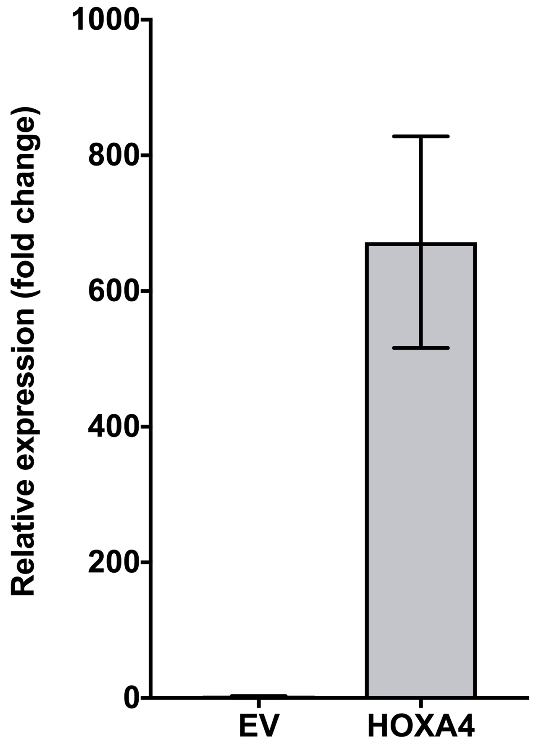

Supplementary Figure 6: Overexpression of HOXA4 in transduced Raji cells. Relative expression of HOXA4 in Raji cells following transduction with a lentiviral vector with the *HOXA4* gene (‘HOXA4’) or empty vector (‘EV’), assessed by qPCR using the $\Delta\Delta$Ct method and *GAPDH* as an endogenous control. The results shown are the product of one qPCR experiment performed in triplicate. Mean values are displayed, with error bars corresponding to standard deviation.

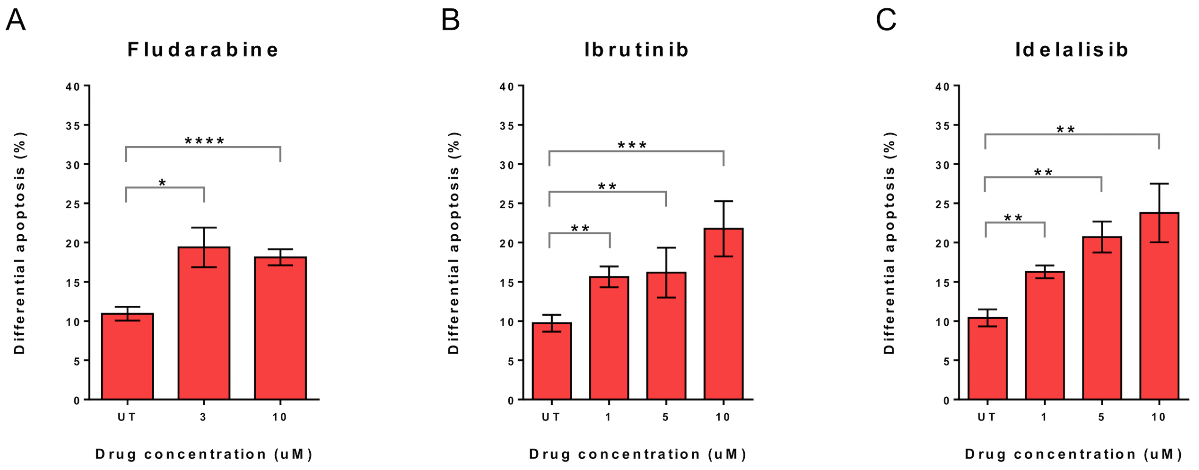

Supplementary Figure 7: Differential apoptosis between *HOXA4*-expressing and control Raji cells following drug exposure. The difference in apoptosis between *HOXA4*-expressing cells and empty vector (EV) controls in untreated (UT) and drug-exposed cells following exposure to fludarabine (A), ibrutinib (B) and idelalisib (C). Apoptosis was measured 48 hours after drug treatment by detection of Annexin V by flow cytometry. Significance relative to untreated cells is indicated (* = p<0.05; ** = p<0.01; *** = p<0.005; **** = p<0.001).
